# Metatranscriptomic analysis revealed *Prevotella* as a potential biomarker of oropharyngeal microbiomes in SARS-CoV-2 infection

**DOI:** 10.3389/fcimb.2023.1161763

**Published:** 2023-06-02

**Authors:** Sifen Lu, Yongzhao Zhou, Ya Hu, Jing Wang, Honghao Li, Yifei Lin, Denian Wang, Jinghong Xian, Shengmei Zhao, Jinmin Ma, Zhongyi Zhu, Shengying Yang, Qinghui Meng, Yulin Kang, Bojiang Chen, Weimin Li

**Affiliations:** ^1^ Precision Medicine Key Laboratory of Sichuan Province and Precision Medicine Center, West China Hospital, Sichuan University, Chengdu, Sichuan, China; ^2^ Department of Integrated Care Management Center, Frontier Science Center of Disease Molecular Network, West China Hospital, Sichuan University, Chengdu, China; ^3^ Center of Infectious Diseases, West China Hospital, Sichuan University, Chengdu, China; ^4^ Department of Hospital Management, West China Hospital, Sichuan University, Chengdu, China; ^5^ Department of Clinical Research Management, West China Hospital, Sichuan University, Chengdu, China; ^6^ Beijing Genomics Institution (BGI)-PathoGenesis Pharmaceutical Technology, Beijing Genomics Institution (BGI)-Shenzhen, Shenzhen, China; ^7^ Department of Computer and Software, Jincheng College of Chengdu, Chengdu, China; ^8^ Beijing Milu Ecological Research Center, Beijing Research Institute of Science and Technology, Beijing, China; ^9^ Institute of Environmental Information, Chinese Research academy of Environmental Sciences, Beijing, China

**Keywords:** COVID-19, *Prevotella*, metatranscriptomic sequencing, oropharyngeal microbiome, SARS-CoV-2 infection, sphingolipid metabolism

## Abstract

**Background and objectives:**

Disease severity and prognosis of coronavirus disease 2019 (COVID-19) disease with other viral infections can be affected by the oropharyngeal microbiome. However, limited research had been carried out to uncover how these diseases are differentially affected by the oropharyngeal microbiome of the patient. Here, we aimed to explore the characteristics of the oropharyngeal microbiota of COVID-19 patients and compare them with those of patients with similar symptoms.

**Methods:**

COVID-19 was diagnosed in patients through the detection of severe acute respiratory syndrome coronavirus 2 (SARS-CoV-2) by quantitative reverse transcription polymerase chain reaction (RT-qPCR). Characterization of the oropharyngeal microbiome was performed by metatranscriptomic sequencing analyses of oropharyngeal swab specimens from 144 COVID-19 patients, 100 patients infected with other viruses, and 40 healthy volunteers.

**Results:**

The oropharyngeal microbiome diversity in patients with SARS-CoV-2 infection was different from that of patients with other infections. *Prevotella* and *Aspergillus* could play a role in the differentiation between patients with SARS-CoV-2 infection and patients with other infections. *Prevotella* could also influence the prognosis of COVID-19 through a mechanism that potentially involved the sphingolipid metabolism regulation pathway.

**Conclusion:**

The oropharyngeal microbiome characterization was different between SARS-CoV-2 infection and infections caused by other viruses. *Prevotella* could act as a biomarker for COVID-19 diagnosis and of host immune response evaluation in SARS-CoV-2 infection. In addition, the cross-talk among *Prevotella*, SARS-CoV-2, and sphingolipid metabolism pathways could provide a basis for the precise diagnosis, prevention, control, and treatment of COVID-19.

## Introduction

The coronavirus disease 2019 (COVID-19) pandemic is a global public health crisis with high mortality rates. This disease is caused by the severe acute respiratory syndrome coronavirus 2 (SARS-CoV-2). Viral infection starts from the upper respiratory tract (URT) and may eventually induce pulmonary disease. In addition, the main microorganism found in the lower respiratory tract of COVID-19 patients originate from related microorganisms in the URT (Shen et al., 2020; Sulaiman et al., 2021). Therefore, confirming the microecological variations in the URT is necessary in COVID-19 patients.

URT is a constitutive element of the anterior nares, nasal cavity, sinuses, nasopharynx, eustachian tube, middle ear cavity, oral cavity, oropharynx, and larynx. A previous study reported that oropharyngeal microbiome alterations could influence COVID-19 severity, by affecting the inflammatory response (Ma et al., 2021). In addition, the oropharyngeal microbiome could affect other respiratory viruses, such as the H7N9 avian influenza (Lu et al., 2017). Some of the earliest public health messages around COVID-19 mentioned that similar symptoms could be related with severe cases of the flu (Dixon et al., 2021). Therefore, distinguishing the characteristics of the oropharyngeal microbiome between patients with COVID-19 and patients with similar symptoms is necessary for the precise prevention and control of COVID-19.

Here, we utilized metatranscriptomic sequencing analyses to conduct a comprehensive investigation on the oropharyngeal microbiome differences in patients with COVID-19 and in patients with similar symptoms. A total of 144 patients with COVID-19, 100 patients infected with other viruses, and 40 healthy individuals were included in this study. We found that *Prevotella* could influence the prognosis of COVID-19. Our study is the first to uncover the potential mechanism of *Prevotella* in SARS-CoV-2 infection.

## Material and methods

### Study design

In this study, we recruited a total of 144 patients infected with COVID-19 (Pos), 100 patients infected with other viruses (Sus), and 40 healthy volunteers (Ctr). The diagnosis of COVID-19 was performed based on the Diagnosis and Treatment Scheme for COVID-19, released by the National Health Commission of China (Version 7) (Shen et al., 2020; Wu et al., 2020). Patients infected with other viruses had similar symptoms as COVID-19 patients; however, SARS-CoV-2 was not detected in their clinical specimens. The inclusion criteria for patients infected with other viruses included positive clinical manifestations but negative IgM antibodies or RNA for SARS-CoV-2. According to specific guidelines (Shen et al., 2020; Wu et al., 2020), the COVID-19 group was further categorized into four severity levels, such as mild (11 cases), moderate (102 cases), severe (13 cases), and critical (18 cases).

This study was approved by the Medical Ethical Committee of West China Hospital of Sichuan University (No. 2020 [100], No. 2020 [193], and No.2020 [267]). Written informed consent complying with the Declaration of Helsinki was obtained from all participants or their legally authorized surrogates.

### Sample collection

Patients were recruited at the West China Hospital of Sichuan University and its allied hospital between February 2020 and June 2020. The procedure for the oropharyngeal swab sample collection was performed according to a video of the standard procedure published by the Chinese Society of Laboratory Medicine (Wang et al., 2020). Briefly, we used a sterile flock swab to collect a sample by wiping the back wall of the pharynx with moderate force and avoiding touching the tongue. To prevent contamination, disposable sterile gloves were used throughout the sample collection process. To assess any potential sample contamination, six additional blank control swabs were included. The 290 collected samples were immediately stored in sterile tubes containing 2 ml of viral transport medium. All the samples were transported to the certified SARS-CoV-2 nucleic acid testing laboratory in a mobile refrigerator and frozen at −80°C. Biological hazard labels were placed on the transport containers and packaging materials.

### RNA extraction

To ensure viral inactivation, all samples were placed in a water bath and heated at 56°C for 45 min. Then, 10 μl protease K (10 mg/ml) and 10 μl carrier RNA (1 mg/ml) were added to each sample tube (1.5 ml), before adding 200 or 400 μl of sample (from the virus-inactivated storage tube). Next, the total RNA of the 290 samples was extracted using the Concert Viral RNA kit (RC1005: Concert Biotech, Xiamen, China) with an HF16 nucleic acid purification instrument, according to the manufacturer’s guidelines.

### Metatranscriptomic library construction and sequencing

After extraction, a standard RNA-seq library was built, using a next-generation sequencing (NGS) library construction kit (Genskey 1906, Beijing, China). Using specific fragment enzyme reactions, RNA was fragmented to suitable lengths for sequencing. After RNA fragmentation, reverse transcription was performed, followed by the synthesis of the complementary cDNA strand. Index adapters were ligated to both ends of the cDNA fragments. Ligation products were purified with DNA clean beads and then amplified through a PCR reaction with 13 cycles. Library concentration was determined using a Qubit Fluorometer (Thermo Fisher Scientific, Shanghai, China). We produced a series of 75 bp single-end sequencing libraries with approximately 20 million reads per sample, using the NextSeq 500 High Output Kit (75 cycles) and an Illumina NextSeq 500 platform (Illumina, Inc., San Diego, CA, United States) at Genskey Gene Company (Beijing, China).

### RT-qPCR assay

The target genes of the real-time-quantitative PCR (RT-qPCR) reaction for SARS-COV-2 detection included the open reading frames of 1ab (ORF1ab) and of the nucleocapsid protein (N). Both were amplified using a RT-PCR kit (Sansure Biotech Inc., China) with a real-time PCR thermal cycler (ABI 7500 system, Applied Biosystems instruments, USA). The primers used were the following: SARS-COV-2_ORF1ab-F:5′-CCCTGTGGGTTTTACACTTAA-3′, SARS-COV-2_ORF1ab-R:5′-ACGATTGTGCATCAGCTGA-3′, SARS-COV-2_ORF1ab-P:5′-FAM-CCGTCTGCGGTATGTGGAAAGGTTATGG-BHQ1-3′, SARS-COV-2_N-F: 5′-GGGGAACTTCTCCTGCTAGAAT-3′, SARS-COV-2_N-R: 5′- CAGACATTTTGCTCTCAAGCTG-3′, and SARS-COV-2_N-P: 5′-FAM-TTGCTGCTGCTTGACAGATT-TAMRA-3′.

When reaction products were positive for both the ORF1ab and N genes (cycle threshold [Ct] < 37), SARS-COV-2 diagnosis was considered positive. When both were negative (with no Ct value or Ct ≥ 40), SARS-COV-2 diagnosis was considered negative.

### Bioinformatics analyses

First, we used the fastp software (parameters: -q 15 –u 40 -l 50, version:0.19.5) (Chen et al., 2018) to filter low-quality reads and remove adapters. To remove low-complexity reads from raw data, we used Komplexity (parameters: -t 0.4, version: Nov-2019) (Clarke et al., 2019). Clean reads were then mapped to the Ensembl 84 (GRCh38) human reference genome to remove host sequences using HISAT2 (version 2.1.0) with default parameters (Kim et al., 2015). Read counts before and after human transcript alignment are shown in **Supplementary Table S4**. Next, we used Kraken2 to annotate taxonomic classifications for unmapped reads (version 2.0.9, parameters: –threads 24 –confidence 0.1) (Wood et al., 2019). For this purpose, we used a self-built database, containing all complete genomes from the NCBI Refseq database, including the SARS-COV-2 reference NC_045512.2. To establish a classification database for Kraken2 (k=35, ℓ=31), only genomes from archaea, bacteria, fungi, protozoa, and viruses were retained. Taxonomic abundance was calculated using Bracken (version 2.5, parameters: -r 75 -l G, S -t 0) (Lu et al., 2017). For subsequent analysis, microorganisms needed to simultaneously meet all the following criteria (1): belong to bacterial, fungal, or viral species (2); have 10-fold or higher filtered reads per million (RPM), in comparison with the negative control (3); no batch effect should be detected; and (4) no known contamination should be detected. Moreover, (5) only sequences of microbes known to infect humans should be selected. In order to obtain *Prevotella* gene sequences, first, unmapped reads were assembled using SPAdes (version: 3.13.0) with default parameters, followed by the removal of scaffolds under 150 bp. Then, the MetaGeneMark tool (Zhu et al., 2010) was used to predict protein-coding genes from the above filtered scaffolds. Next, sequences of these predicted genes were aligned with the sequences of *Prevotella* from the NCBI Refseq database, using the basic local alignment search tool (BLAST) with an e-value=1e-7 as a cutoff criterion (Ye et al., 2006). Sequences with more than 80% aligned lengths were kept. Kyoto Encyclopedia of Genes and Genomes (KEGG) pathway analysis of *Prevotella* genes was performed using the KEGG Orthology Based Annotation System (KOBAS v3; http://kobas.cbi.pku.edu.cn/kobas3) online tool (Xie et al., 2011). The *p-*values were corrected using the Benjamini and Hochberg procedure (Benjamini and Hochberg, 1995). A corrected *p*-value of ≤ 0.05 was considered to be statistically significant.

### Statistical analyses

Statistical analyses were performed using the R software (version 3.5.1). The ratio between the abundance of a specific genus in a sample and the sum of all genus abundances in the same sample was used to quantify the relative abundance of each genus in the oropharyngeal microbiome. The vegan function of the R package was used to analyze the alpha diversity. Differences between groups were analyzed using the Mann–Whitney test or the Kruskal–Wallis test or analysis of variance. False discovery rate (FDR) values were estimated using the Benjamini–Yekutieli procedure to control for multiple testing. Principal coordinate analysis (PCoA) was based on Bray–Curtis dissimilarity (weighed) distance matrices. We used analysis of similarities (ANOSIM) to determine significant differences between two or more groups of sampling units. Finally, the Spearman correlation method was used to analyze the correlation between microbes and clinical indices, being a correlation coefficient of >0.300 considered acceptable. The relative abundances in the oropharyngeal microbiome between different groups were calculated using the Wilcoxon rank-sum test. *p*-values of <0.05 (*), <0.01 (**), and <0.001 (***) were considered statistically significant.

## Results

### Clinical characteristics of the included participants

The demographics and clinical characteristics of the 284 participants are shown in **Table 1**. In the Pos group, 22.9% (33/144) participants were older than 60 years; 58.3% (84/144) participants were male; and 7.6% (11/144), 70.8% (102/144), 9.0% (13/144), and 12.5% (18/144) of the 144 participants in the Pos group were assigned with mild, moderate, severe, and critical, respectively. Of the patients, 45.1% (65/144) have used antibiotics. The average alanine transaminase (ALT) was 22.5, the average high-sensitivity C-reactive protein (CRP) test result was 8.75, the average lymphocytes (LYMPH) test result was 24.2, the average monocytes (MONO) test result was 6.2, the average neutrophils (NEUT) test result was 66.1, and the average procalcitonin (PCT) test result was 0.03. Regarding treatment prognosis, 56.9% (82/144) patients have recovered, 16.7% (24/144) have improved, 20.1% (29/144) remained in the hospital, 0.69% (1/144) patient died, and 5.56% (8/144) patients had no relevant information available. Of note, age was significantly different among the three groups (*p* < 0.001; **Table 1**).

**Table 1:** Demographics and clinical characteristics of the participants.

Characteristics	Pos (n=144)	Sus (n=100)		Ctr (n=40)	F-value	Pr (>F)
**Age**					8.472	< 0.001
1–20 years	7 (4.9%)	7 (7.0%)		3 (7.5%)		
20–40 years	45 (31.3%)	57 (57.0%)		14 (35.0%)		
40–60 years	59 (40.9%)	23 (23.0%)		14 (35.0%)		
>60 years	33 (22.9%)	13 (13.0%)		9 (22.5%)		
**Gender**					0.602	0.548
Female (%)	60 (41.7%)	40 (40.0%)		20 (50.0%)		
Male (%)	84 (58.3%)	60 (60.0%)		20 (50.0%)		
Smoking history						
Ongoing	12 (8.3%)	7		NA		
Never	123 (85.4%)	85		NA		
Ever	9 (6.3%)	8		NA		
Disease severity						
Mild	11 (7.6%)	NA		NA		
Moderate	102 (70.8%)	NA		NA		
Severe	13 (9.0%)	NA		NA		
Critical	18 (12.5%)	NA		NA		
Length of stay						
≥14 days	121 (84.0%)	NA		NA		
≥28 days	47 (32.6%)	NA		NA		
No relevant information	8 (5.6%)	NA		NA		
Symptoms						
Cough	93 (64.6%)	86 (86%)		NA		
Fever	89 (61.8%)	100 (100%)		NA		
Sputum	54 (37.5%)	58 (58%)		NA		
Fatigue	27 (18.8%)	20 (20%)		NA		
Sore throat	15 (10.4%)	56 (56%)		NA		
Muscle and joint pain	15 (10.4%)	34 (34%)		NA		
Headache	14 (9.7%)	43 (43%)		NA		
Gastrointestinal symptoms	12 (8.3%)	8 (8%)		NA		
Dyspnea	9 (6.3%)	3 (3%)		NA		
Runny nose	8 (5.6%)	24 (24%)		NA		
Conjunctivitis	2 (1.4%)	0		NA		
Comorbidities						
Yes	79 (54.9%)	56 (56%)		NA		
No	65 (45.1%)	44 (44%)		NA		
Antibiotics use						
Yes	65 (45.1%)	16 (16%)		NA		
No	79 (54.9%)	84 (84%)		NA		
Clinical indicators value						
Hb (g/L)	146 (60–182)	NA		NA		
PLT (×10^9^/L)	175 (63–368)	NA		NA		
NEUT (%)	66.1 (3.1–95.4)	NA		NA		
LYMPH (%)	24.2 (1.8–53.1)	NA		NA		
MONO (%)	6.2 (0.11–20.5)	NA		NA		
EO (%)	0.1 (0.0–3.8)	NA		NA		
BASO (%)	0.1 (0.0–1.3)	NA		NA		
HCT (L/L)	43.1 (0.2–51.5)	NA		NA		
PCT (ng/ml)	0.03 (0.01–109)	NA		NA		
CRP (mg/L)	8.75 (0.1–119.2)	NA		NA		
ALT (IU/L)	22.5 (3–151)	NA		NA		
AST (IU/L)	23 (9.8–502)	NA		NA		
TBil (μmol/L)	8.4 (2.0–45.9)	NA		NA		
CREA (μmol/L)	69 (14–969)	NA		NA		
Outcome						
Recovery	82 (56.9%)	NA		NA		
Improved	24 (16.7%)	NA		NA		
Continue to stay	29 (20.1%)	NA		NA		
Death	1 (0.69%)	NA		NA		
No relevant information	8 (5.56%)	NA		NA		

NA, not available.

### Differences in the characterization of oropharyngeal bacteria between infection with SARS-CoV-2 and infection with other viruses

**Figure 1:**
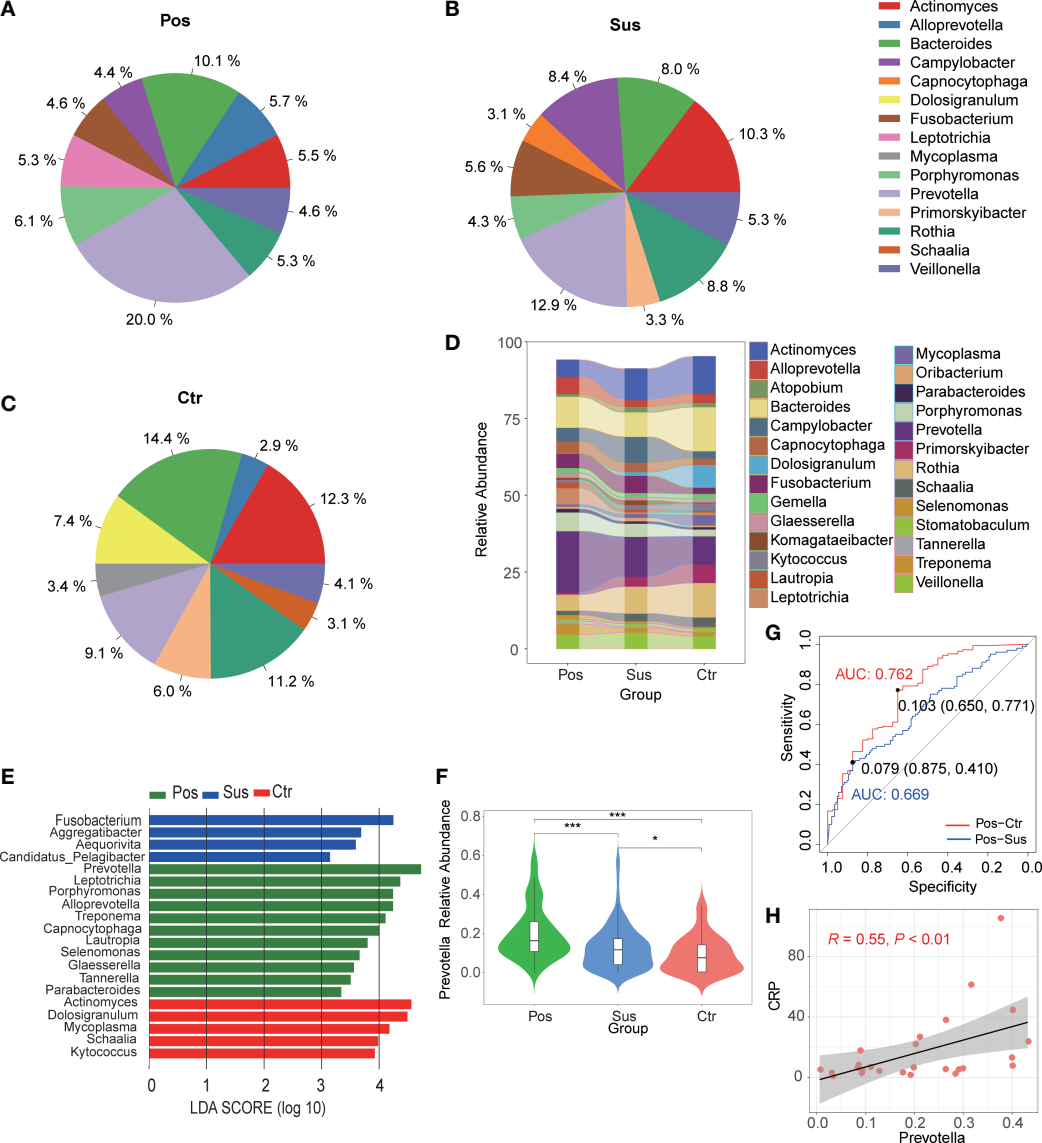
Oropharyngeal bacterial characterization among Pos, Sus, and Ctr. Average compositions of relative abundance of the top 10 bacterial genera for **(A)** Pos, **(B)** Sus, and **(C)** Ctr. **(D)** Alterations of all the top 20 bacterial genera among the three groups. **(E)** Differentially abundant bacteria among Pos, Sus, and Ctr. **(F)** The relative abundance of *Prevotella* among the three groups. **(G)** The ROC curve of *Prevotella* between Pos and Sus and between Pos and Ctr. **(H)** The correlation between the abundance of *Prevotella* and the value of CRP. LDA score was represented as the degree of difference among the three groups. The *p*-values were confirmed by the Wilcoxon rank-sum test. LDA, linear discriminant analysis; ROC, receiver operating characteristic; AUC, area under curve; CRP, C-reactive protein. p-values of <0.05 (*), <0.01 (**), and <0.001 (***) were considered statistically significant.

Overall, 780 bacterial genera were identified in the 284 studied samples. The relative abundance of the top 10 bacterial profiles in the Pos, Sus, and Ctr samples are shown in **Figures 1A–C**, respectively. Apparently, the relative abundance of *Actinomyces*, *Bacteroides*, *Campylobacter*, *Fusobacterium*, *Prevotella*, *Primorskyibacter*, and *Rothia* obviously changed among the Pos, Sus, and Ctr groups (**Figure 1D**). In addition, among the above seven bacterial genera, the lefse result showed that *Prevotella* was enriched in the Pos group, *Fusobacterium* was enriched in the Sus group, and *Actinomyces* was enriched in the Ctr group (**Figure 1E**). Only the abundance of *Prevotella* significantly changed among the Pos, Sus, and Ctr groups (*p* < 0.05; **Supplementary Table S1** **;** **Figure 1F**). Then, we evaluated whether the abundance of *Prevotella* could accurately differentiate between the Pos and Sus groups and between the Pos and Ctr groups. As a result, *Prevotella* showed an AUC of 0.669 between the Pos and Sus groups and an AUC of 0.762 between the Pos and Ctr groups (**Figure 1G**). In addition, we evaluated whether *Prevotella* could play a role in host immune response evaluation. The abundance of *Prevotella* in the Pos group was significantly and positively correlated with the value of CRP R = 0.55, *p* < 0.01; **Figure 1H**).

### Differences in the characterization of oropharyngeal fungi between infection with SARS-CoV-2 and infection with other viruses

**Figure 2:**
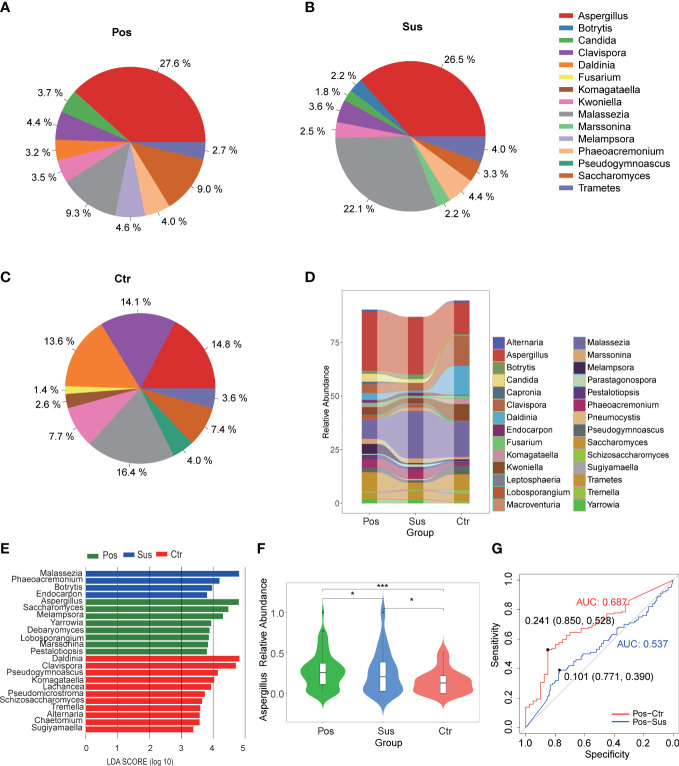
Oropharyngeal fungal characterization among Pos, Sus, and Ctr. Average compositions of relative abundance of the top 10 fungal genera for **(A)** Pos, **(B)** Sus, and **(C)** Ctr. **(D)** Alterations of all the top 20 fungal genera among the three groups. **(E)** Differentially abundant fungi among Pos, Sus, and Ctr. **(F)** The relative abundance of *Aspergillus* among the three groups. **(G)** The ROC curve of *Aspergillus* between Pos and Sus and between Pos and Ctr. LDA score was represented as the degree of difference among the three groups. The *p*-values were confirmed by the Wilcoxon rank-sum test. LDA, linear discriminant analysis; ROC, receiver operating characteristic; AUC, area under curve. p-values of <0.05 (*), <0.01 (**), and <0.001 (***) were considered statistically significant.

Overall, 177 genera of fungi were identified in the 284 studied samples. The relative abundance of the top 10 fungal profiles in the Pos, Sus, and Ctr samples are shown in **Figures 2A–C**. Specifically, the relative abundance of *Aspergillus*, *Malassezia*, and *Daldinia* obviously changed among the Pos, Sus, and Ctr groups (**Figure 2D**). In addition, among the above three fungi, the lefse result showed that *Aspergillus* was enriched in the Pos group, *Malassezia* was enriched in the Sus group, and *Daldinia* was enriched in the Ctr group (**Figure 2E**). Only *Aspergillus* significantly varied among the Pos, Sus, and Ctr groups (*p* < 0.05; **Supplementary Table S2** **;** **Figure 2F**). Then, we evaluated whether *Aspergillus* could accurately differentiate among the Pos and Sus and the Pos and Ctr groups. As a result, *Aspergillus* had an AUC of 0.537 between the Pos and Sus groups and an AUC of 0.687 between the Pos and Ctr groups (**Figure 2G**). In addition, we evaluated whether *Aspergillus* could play a role in host immune response evaluation. However, *Aspergillus* abundance in the Pos group showed no significant correlation with any clinical indicators related to host immune response.

### Differences in the characterization of oropharyngeal viruses between infection with SARS-CoV-2 and infection with other viruses

**Figure 3:**
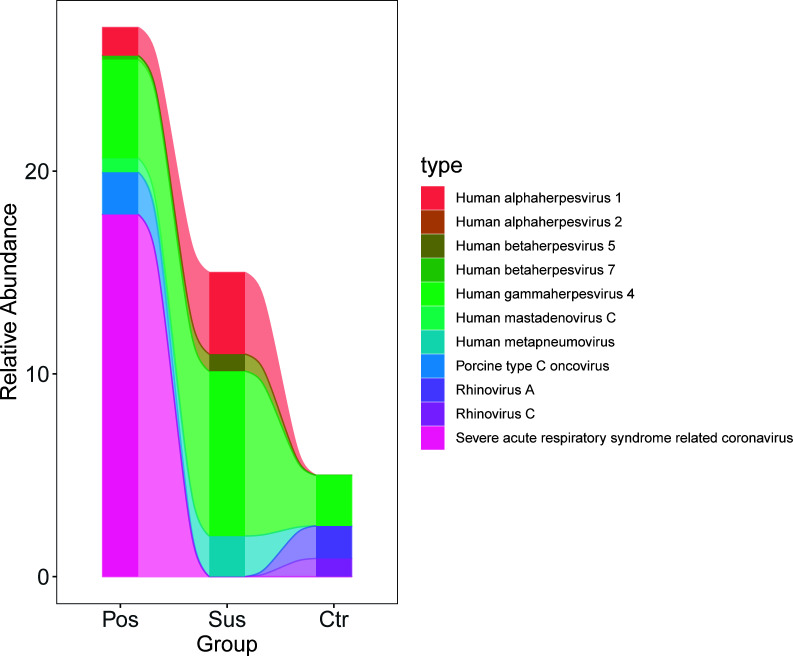
Oropharyngeal viral characterization among Pos, Sus, and Ctr. The relative abundance changes of 11 oropharyngeal viral species (including SARS-CoV-2) among Pos, Sus, and Ctr.

The relative abundance of SARS-CoV-2 in the Pos samples was significantly higher than that in the Sus and Ctr samples (**Figure 3** **;** **Supplementary Table S3** **;** *p* < 0.05). However, the relative abundance of *Porcine type-C oncovirus*, *Human alphaherpesvirus 1*, *Human betaherpesvirus 5*, *Human gammaherpesvirus 4*, *Human mastadenovirus C*, *Human metapneumovirus*, *Rhinovirus A*, and *Rhinovirus C* showed no significant differences among the Pos, Sus, and Ctr groups (**Figure 3** **;** **Supplementary Table S3** **;** *p* > 0.05).

### Differences in the characterization of oropharyngeal microbiome diversity between infection with SARS-CoV-2 and infection with other viruses

**Figure 4:**
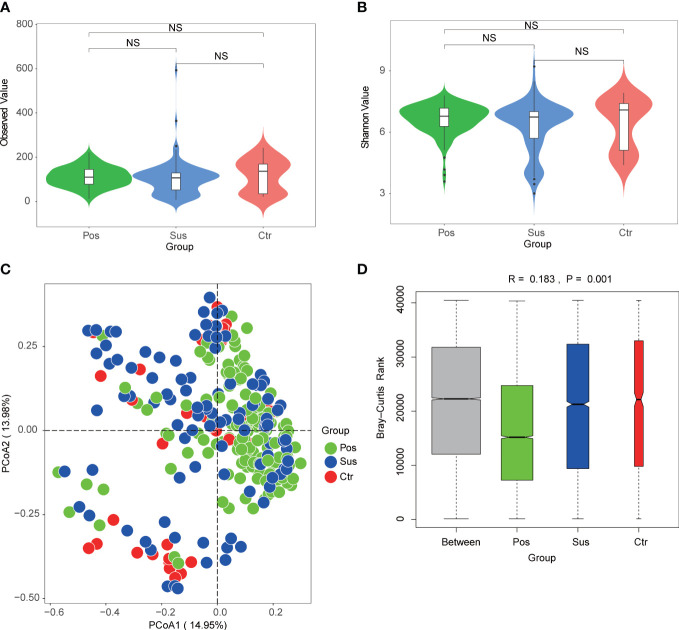
Oropharynx microbial diversity characterization among Pos, Sus, and Ctr. Alpha diversity among Pos, Sus, and Ctr, including **(A)** Observed index (NS: *p*>0.05) and **(B)** Shannon index (NS: *p*>0.05). **(C)** Principal coordinate analysis (PCoA) based on Bray–Curtis (weighed) distance showed obvious changes in beta diversity among Pos, Sus, and Ctr. The colors represent three different groups. PCoA1 and PCoA2 represent the top 2 principal coordinates that captured most of the diversity. The fraction of diversity captured by the coordinate is valued as a percentage. **(D)** The analysis of similarities (ANOSIM) based on Bray–Curtis (weighed) distance (R > 0 and *p* < 0.05). The R means the statistical value of ANOSIM; y-axis value means Bray–Curtis rank; and 999 was used for the permutations. NS, not significant.

To explore the differences in microbial richness and community structure, we compared the alpha and beta diversities of samples from patients infected with SARS-CoV-2 and patients infected with other viruses. As a result, minimal alpha diversity changes were observed among the Pos, Sus, and Ctr groups, including the Observed, Shannon (**Figures 4A, B**; *p* > 0.05), Chao1, and Simpson indices (**Supplementary Figures S1A, B**; *p* > 0.05). In addition, the beta diversity results showed that the Pos group was mainly separated from the Ctr and Sus groups (**Figure 4C**). The analysis of similarities (ANOSIM) plot reveals that the intergroup difference was greater than the intra-group difference (**Figure 4D**, R= 0.183, *p* = 0.001).

### *Prevotella* played a potential role in SARS-CoV-2 infection

**Figure 5:**
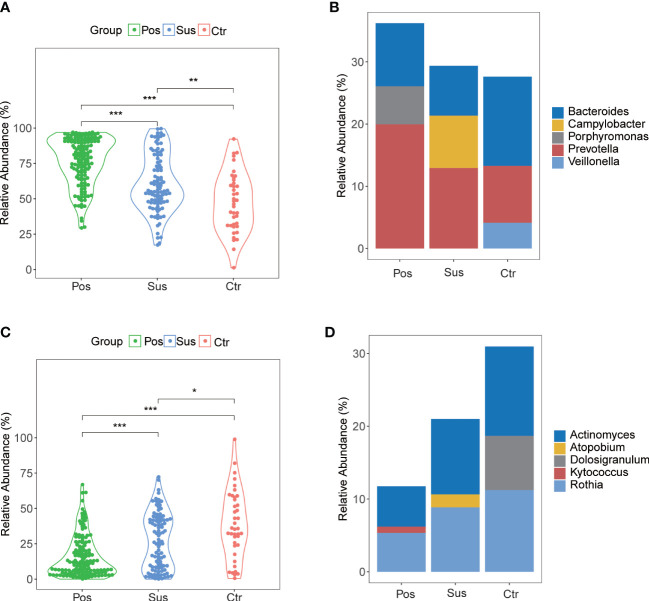
Gram-negative and Gram-positive bacteria among Pos, Sus, and Ctr. **(A)** The relative abundance of Gram-negative in Pos was a little greater than that in Sus (*p* < 0.001). **(B)** The major bacteria contributing to this phenotype were *Prevotella* and *Bacteroides*. **(C)** The relative abundance of Gram-positive in Pos was lower than that in Sus (*p* < 0.001). **(D)** The major bacteria contributing to this phenotype were *Rothia* and *Actinomyces.* The *p*-values were confirmed by the Wilcoxon rank-sum test. p-values of <0.05 (*), <0.01 (**), and <0.001 (***) were considered statistically significant.

**Figure 6:**
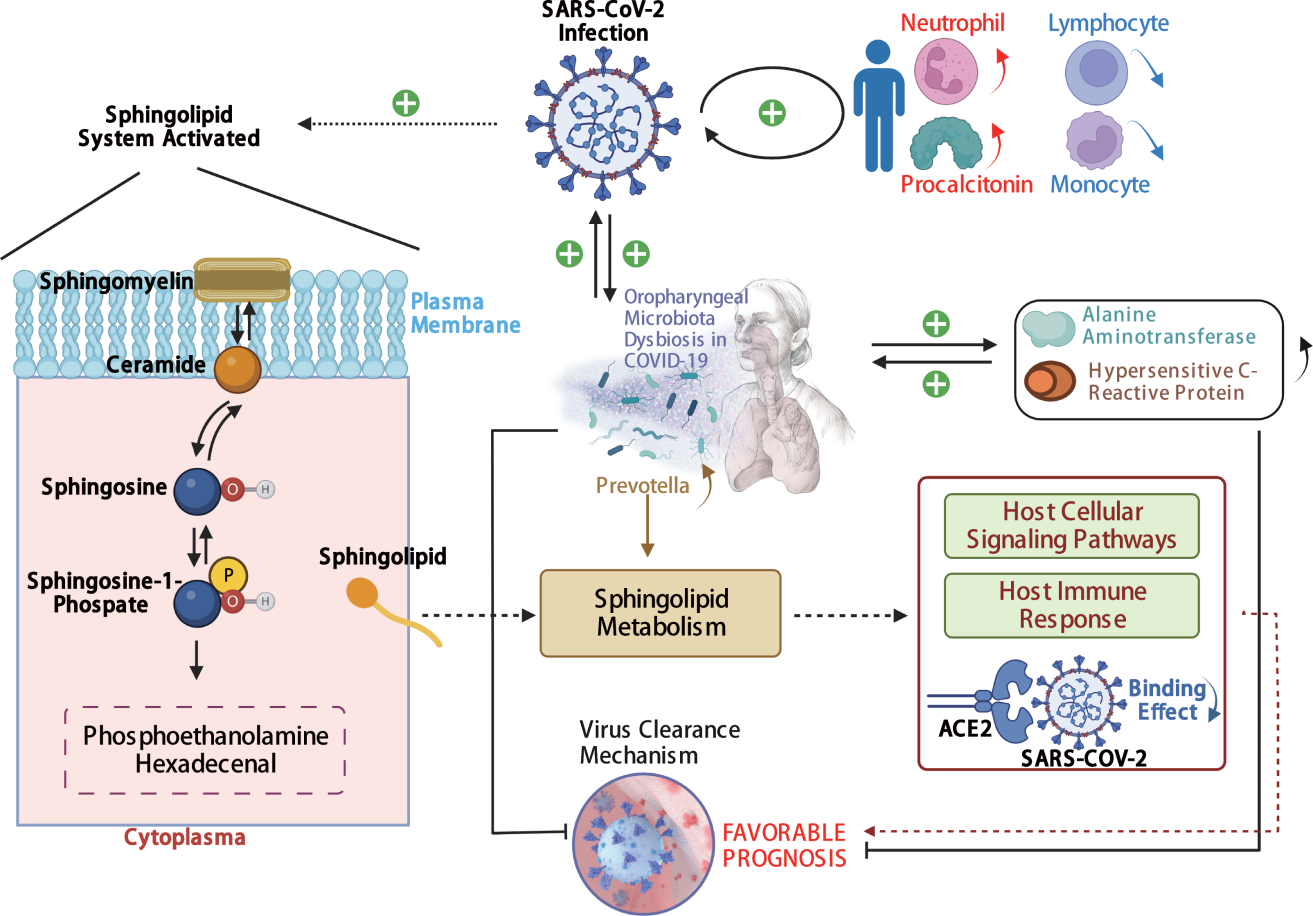
The potential mechanism of *Prevotella* involved in the SARS-CoV-2 infection. The solid arrows are derived from the findings of this study, and the dashed arrows are derived from the findings of previous reports.

In our study, the abundance and diversity of the oropharyngeal microbiome in patients with SARS-CoV-2 infection were different from those in the patients infected with other viruses. Therefore, we further analyzed the potential function of the oropharyngeal microbiome in SARS-CoV-2 infection in order to uncover its potential mechanism in the infection. As a result, the relative abundance of Gram-negative bacteria (**Figure 5A**; *p* < 0.001) in the Pos group was slightly higher than that in the Sus and Ctr groups. The major bacteria contributing to this phenotype were *Prevotella* and *Bacteroides* (**Figure 5B**). The relative abundance of Gram-positive bacteria in the Pos group was significantly lower than that in the Sus and Ctr groups (**Figure 5C**; *p* < 0.001). The major bacteria contributing to this phenotype were *Rothia* and *Actinomyces* (**Figure 5D**). These results indicate that the oropharyngeal bacteria in samples with SARS-CoV-2 infection were mainly Gram-negative, especially *Prevotella*. Then, we attempted to analyze the potential role of *Prevotella*. The relative abundance of *Prevotella* was significantly and positively correlated with the value of some clinical indicators (**Supplementary Figure S2A**), including alanine transaminase (ALT; R = 0.50, *p* < 0.01) and Outcome (R = 0.44, *p* < 0.05). It is worth noting that the higher the Outcome value, the worse the prognosis (1, recovery; 2, improved or continue to stay; and 3, death). In addition, the relative abundance of *Prevotella* was significantly and positively correlated with the number of *Prevotella* genes involved in these pathways (**Supplementary Figure S2B**), including arachidonic acid metabolism (ACM; R = 0.55, *p* < 0.01), galactose metabolism (GM; R = 0.54, *p* < 0.01), isoquinoline alkaloid biosynthesis (IAB; R = 0.50, *p* < 0.01), other glycan degradation (OGD; R = 0.53, *p* < 0.01), sphingolipid metabolism (SM; R = 0.61, *p* < 0.001), and steroid hormone biosynthesis (SHB; R = 0.51, *p* < 0.01). Interestingly, the relative abundance of *Prevotella* showed a positive correlation trend with the relative abundance of SARS-CoV-2 (**Supplementary Figure S3A**; R = 0.028, *p* > 0.05). The values of ALT (R = 0.41, *p* < 0.05) and CRP (R = 0.62, *p* < 0.001) were significantly and positively correlated with the Outcome value (**Supplementary Figure S3B**). The percentage of lymphocytes (LYMPH; R = −0.57, *p* < 0.01) and monocytes (MONO; R = −0.41, *p* < 0.05) were significantly and negatively correlated with the Severity value. Nevertheless, the percentage of neutrophils (NEUT; R = 0.50, *p* < 0.01) and the Procalcitonin value (PCT; R = 0.54, *p* < 0.01) were significantly and positively correlated with the Severity value (**Supplementary Figure S3C**). Notably, the higher the Severity value, the more serious it is (1, mild; 2, moderate; 3, severe; 4, critical). In addition, sphingolipid has been reported to be closely related to SARS-CoV-2 infection (Torretta et al., 2021; Vitner et al., 2022). Based on these results, we deduced the potential mechanism of *Prevotella* in the SARS-CoV-2 infection (**Figure 6**).

## Discussion

Our study was the first to use metatranscriptomic sequencing and analysis to uncover the different composition of the oropharyngeal bacteria, fungi, and viruses between patients infected with SARS-CoV-2 and patients infected with other viruses. This study also reveals the potential roles of the oropharyngeal microbiome in SARS-CoV-2 infection.

First, minimal changes were observed in alpha diversity among the Pos, Sus, and Ctr groups, including the Observed, Shannon, Chao1, and Simpson indices. These observations are consistent with previous findings showing no significant differences in alpha diversity between COVID-19 patients and healthy controls (Miller et al., 2021). The beta diversity results showed that the Pos group was slightly separated from the Ctr and Sus groups. This indicates that the effect of SARS-CoV-2 infection on oropharyngeal microbial community distribution is different from that of other viral infections. This is partially consistent with the findings of a previous report revealing that the pattern of SARS-CoV-2 infection is quite distinct from that of other human pathogenic respiratory viruses, such as the influenza A virus and the respiratory syncytial virus (Simmonds et al., 2021).

Another important aspect to mention is that the relative abundance of *Aspergillus* decreased progressively among the Pos, Sus, and Ctr groups, with an AUC of 0.537 between the Pos and Sus groups and an AUC of 0.687 between the Pos and Ctr groups. This indicated that the abundance of *Aspergillus* could also play a role in distinguishing the infection with SARS-CoV-2 from the infection with other viruses. A previous study also showed that secondary bacterial and fungal coinfections with SARS-CoV-2 could be mainly due to *Aspergillus* (Blaize et al., 2020; Rabagliati et al., 2021). This finding further corroborates our findings.

One of the most exciting findings of our study is that the relative abundance of *Prevotella* varied significantly among COVID-19 patients (Pos), patients infected with other viruses (Sus), and healthy volunteers (Ctr). The AUC of 0.669 between the Pos and Sus groups and the AUC of 0.762 between the Pos and Ctr groups indicated that *Prevotella* could function as a biomarker in distinguishing between patients infected with SARS-CoV-2 and patients infected with other viruses. Previous findings showing that *Prevotella* was the main bacterium in the URT of COVID-19 patients (Wang et al., 2020) and the decreased abundance of *Prevotella* in patients with viral respiratory tract infections (influenza A, influenza B, rhinovirus, metapneumovirus, and respiratory syncytial virus) (Edouard et al., 2018) could support our findings. In addition, the relative abundance of Gram-negative bacteria in the Pos group was slightly higher than that in the Sus and Ctr groups. The major bacteria contributing to this phenotype were *Prevotella* and *Bacteroides*. The oropharyngeal bacteria in samples with SARS-CoV-2 infection are mainly Gram-negative bacteria, especially *Prevotella*. This observation is consistent with previous findings showing that Gram-negative pathogens are the major cause of bacterial pneumonia in critical COVID-19 patients (Dudoignon et al., 2021). More importantly, the abundance of *Prevotella* in the Pos group was significantly and positively correlated with the value of CRP (R = 0.55, *p* < 0.01), which indicated that *Prevotella* could function as a biomarker in host immune response evaluation in SARS-CoV-2 infection. In addition, also the interaction between CRP and the immune response to pathogens has been previously reported (Sproston and Ashworth, 2018), and CRP has been related to the innate immune system (Du Clos, 2000).

Here, the potential mechanism of *Prevotella* involvement in the SARS-CoV-2 infection is illustrated in **Figure 6**. The potential mechanism supports that *Prevotella* can participate in the immune homeostasis of the lower respiratory tract (Huffnagle et al., 2017). *Prevotella* species are anaerobic Gram-negative bacteria that can promote inflammatory disease features. Pulmonary inflammation, which is associated with the accumulation of the pulmonary microbiome with *Prevotella* (Segal et al., 2013; Yuan et al., 2022), was more severe in infections with SARS-CoV-2 than in infections with other viruses. Another mechanism involved was the sphingolipid metabolic pathway. This pathway modulates viral replication, affects host cellular signaling pathways needed for viral propagation, and regulates host innate and adaptive immune responses to the infection (Vijayan and Hahm, 2014). In addition, sphingosine could inhibit the interaction of SARS-CoV-2 with its ACE2 receptor (Törnquist et al., 2021). Humans with oropharyngeal microbiome dysbiosis are vulnerable to infection by SARS-CoV-2, thereby increasing inflammation. On the other hand, SARS-CoV-2 infection changed the balance of *Prevotella* and increased inflammation. Our results showed that *Prevotella* was enriched in the Pos group. We derived that SARS-CoV-2 elevation may induce the increase in *Prevotella*, and its genes could be involved in the sphingolipid metabolic pathway, and the sphingosine in turn may inhibit the increase in SARS-CoV-2. Therefore, *Prevotella* could participate in the sphingolipid metabolism pathway, influencing COVID-19 prognosis.

Our study has some limitations. First, incomplete clinical data prevented us from comparing the variants of participant characteristics and showing the correlation between the abundance of *Prevotella* and the value of CRP in the Sus group. Second, antibiotic effects, which could be a confounding factor on the respiratory tract flora, were not analyzed in our study. Third, we have not achieved any relevant explanation about the porcine type-C oncovirus because it was reported to be a pathogen associated with porcine infection (Ye et al., 2022), and its effects on human beings lack relevant studies. Fourth, the relative abundance of *Prevotella* showed a positive correlation trend with the relative abundance of SARS-CoV-2; however, the result was not statistically significant, having a low R-value. As such, the relationship between *Provotella* and the disease outcome had to be analyzed by univariate methods. Fifth, we explained the potential pathway through which *Prevotella* plays a role in COVID-19; however, there is lack of biological experiments and lack of the overall functional factors. Efforts would be made to explore the overall molecular mechanisms of the oropharyngeal microbiome (especially *Prevotella*) effects in the development of COVID-19.

To summarize, this study revealed the differences in the oropharyngeal microbiome between patients infected with SARS-CoV-2 and patients infected with other viruses. Furthermore, and for the first time, our analyses explored the potential mechanism of the oropharyngeal microbiome in SARS-CoV-2 infection. We found distinct oropharyngeal microbiome diversity in patients infected with SARS-CoV-2 and in patients infected with other viruses. *Prevotella* and *Aspergillus* could play a role in distinguishing the two types of infections. *Prevotella* could influence the prognosis of COVID-19 through its role in sphingolipid metabolism regulation.

In conclusion, we provided evidence that the oropharyngeal microbial characteristics of the SARS-CoV-2 infection are different from those of the infection with other viruses. *Prevotella* could function as a biomarker of COVID-19 and of host immune response evaluation in SARS-CoV-2 infection. The cross-talk among *Prevotella*, SARS-CoV-2, and sphingolipid metabolism pathway could provide a basis for the precise diagnosis, prevention, control, and treatment of COVID-19.

## Data availability statement

The datasets presented in this study can be found in online repositories. The names of the repository/repositories and accession number(s) can be found below: CNSA (https://db.cngb.org/cnsa/) of CNGBdb,CNP0001393.

## Ethics statement

The studies involving human participants were reviewed and approved by Medical Ethical Committee of West China Hospital. The patients/participants provided their written informed consent to participate in this study.

## Author contributions

WL, BC, and YK contributed substantially to the design of the study. SL, YZ, YH, and JW contributed substantially to the understanding of data and wrote the manuscript. DW, JX, and SZ undertook clinical sample collection. SL, YZ, YH, JW, JM, ZZ, and SY contributed substantially to the data analysis. HL and YL helped to improve the expression for important intellectual content. WL, BC, and YK revised the manuscript. All authors contributed to the article and approved the submitted version.

## References

[B1] Benjamini Y. Hochberg Y. (1995). Controlling the false discovery rate: a practical and powerful approach to multiple testing. J. R. Stat. society: Ser. B (Methodological) 57 (1), 289–300. doi: 10.1111/j.2517-6161.1995.tb02031.x

[B2] Blaize M. Mayaux J. Nabet C. Lampros A. Marcelin A.-G. Thellier M.. (2020). Fatal invasive aspergillosis and coronavirus disease in an immunocompetent patient. Emerg. Infect. Dis. 26 (7), 1636–1637. doi: 10.3201/eid2607.201603 32343223 PMC7323532

[B3] Chen S. Zhou Y. Chen Y. Gu J. (2018). Fastp: an ultra-fast all-in-one FASTQ preprocessor. Bioinformatics 34 (17), i884–i890. doi: 10.1093/bioinformatics/bty560 30423086 PMC6129281

[B4] Clarke E. L. Taylor L. J. Zhao C. Connell A. Lee J.-J. Fett B.. (2019). Sunbeam: an extensible pipeline for analyzing metagenomic sequencing experiments. Microbiome 7 (1), 46. doi: 10.1186/s40168-019-0658-x 30902113 PMC6429786

[B5] Dixon B. E. Wools-Kaloustian K. K. Fadel W. F. Duszynski T. J. Yiannoutsos C. Halverson P. K.. (2021). Symptoms and symptom clusters associated with SARS-CoV-2 infection in community-based populations: results from a statewide epidemiological study. PloS One 16 (3), e0241875. doi: 10.1371/journal.pone.0241875 33760821 PMC7990210

[B6] Du Clos T. W. (2000). Function of c-reactive protein. Ann. Med. 32 (4), 274–278. doi: 10.3109/07853890009011772 10852144

[B7] Dudoignon E. Caméléna F. Deniau B. Habay A. Coutrot M. Ressaire Q.. (2021). Bacterial pneumonia in COVID-19 critically ill patients: a case series. Clin. Infect. Dis. 72 (5), 905–906. doi: 10.1093/cid/ciaa762 32544219 PMC7337703

[B8] Edouard S. Million M. Bachar D. Dubourg G. Michelle C. Ninove L.. (2018). The nasopharyngeal microbiota in patients with viral respiratory tract infections is enriched in bacterial pathogens. Eur. J. Clin. Microbiol. Infect. Dis. 37 (9), 1725–1733. doi: 10.1007/s10096-018-3305-8 30033505

[B9] Huffnagle G. Dickson R. Lukacs N. (2017). The respiratory tract microbiome and lung inflammation: a two-way street. Mucosal Immunol. 10 (2), 299–306. doi: 10.1038/mi.2016.108 27966551 PMC5765541

[B10] Kim D. Langmead B. Salzberg S. L. (2015). HISAT: a fast spliced aligner with low memory requirements. Nat. Methods 12 (4), 357–360. doi: 10.1038/nmeth.3317 25751142 PMC4655817

[B11] Lu J. Breitwieser F. P. Thielen P. Salzberg S. L. (2017). Bracken: estimating species abundance in metagenomics data. PeerJ Comput. Sci. 3, e104. doi: 10.7717/peerj-cs.104 PMC12016282 40271438

[B12] Lu H.-f. Li A. Zhang T. Ren Z.-g. He K.-x. Zhang H.. (2017). Disordered oropharyngeal microbial communities in H7N9 patients with or without secondary bacterial lung infection. Emerg. Microbes infections 6 (12), e112. doi: 10.1038/emi.2017.101 PMC5750457 29259328

[B13] Ma S. Zhang F. Zhou F. Li H. Ge W. Gan R.. (2021). Metagenomic analysis reveals oropharyngeal microbiota alterations in patients with COVID-19. Signal Transduct. targeted Ther. 6 (1), 191. doi: 10.1038/s41392-021-00614-3 PMC8116522 33986253

[B14] Miller E. H. Annavajhala M. K. Chong A. M. Park H. Nobel Y. R. Soroush A.. (2021). Oral microbiome alterations and SARS-CoV-2 saliva viral load in patients with COVID-19. Microbiol. Spectr. 9 (2), e0005521. doi: 10.1128/Spectrum.00055-21 34643448 PMC8515944

[B15] Rabagliati R. Rodríguez N. Núñez C. Huete A. Bravo S. Garcia P. (2021). COVID-19–associated mold infection in critically ill patients, Chile. Emerg. Infect. Dis. 27 (5), 1454–1456. doi: 10.3201/eid2705.204412 33760726 PMC8084475

[B16] Segal L. N. Alekseyenko A. V. Clemente J. C. Kulkarni R. Wu B. Chen H.. (2013). Enrichment of lung microbiome with supraglottic taxa is associated with increased pulmonary inflammation. Microbiome 1 (1), 19. doi: 10.1186/2049-2618-1-19 24450871 PMC3971609

[B17] Shen Z. Xiao Y. Kang L. Ma W. Shi L. Zhang L.. (2020). Genomic diversity of severe acute respiratory syndrome–coronavirus 2 in patients with coronavirus disease 2019. Clin. Infect. Dis. 71 (15), 713–720. doi: 10.1093/cid/ciaa203 32129843 PMC7108196

[B18] Simmonds P. Williams S. Harvala H. (2021). Understanding the outcomes of COVID-19–does the current model of an acute respiratory infection really fit? J. Gen. Virol. 102 (3), 001545. doi: 10.1099/jgv.0.001545 33331810 PMC8222868

[B19] Sproston N. R. Ashworth J. J. (2018). Role of c-reactive protein at sites of inflammation and infection. Front. Immunol. 9, 754. doi: 10.3389/fimmu.2018.00754 29706967 PMC5908901

[B20] Sulaiman I. Chung M. Angel L. Tsay J.-C. J. Wu B. G. Yeung S. T.. (2021). Microbial signatures in the lower airways of mechanically ventilated COVID-19 patients associated with poor clinical outcome. Nat. Microbiol. 6 (10), 1245–1258. doi: 10.1038/s41564-021-00961-5 34465900 PMC8484067

[B21] Törnquist K. Asghar M. Y. Srinivasan V. Korhonen L. Lindholm D. (2021). Sphingolipids as modulators of SARS-CoV-2 infection. Front. Cell Dev. Biol. 9, 689854. doi: 10.3389/fcell.2021.689854 34222257 PMC8245774

[B22] Torretta E. Garziano M. Poliseno M. Capitanio D. Biasin M. Santantonio T. A.. (2021). Severity of COVID-19 patients predicted by serum sphingolipids signature. Int. J. Mol. Sci. 22 (19), 10198. doi: 10.3390/ijms221910198 34638539 PMC8508132

[B23] Vijayan M. Hahm B. (2014). Influenza viral manipulation of sphingolipid metabolism and signaling to modulate host defense system. Scientifica 2014, 793815. doi: 10.1155/2014/793815 24672735 PMC3920843

[B24] Vitner E. B. Avraham R. Politi B. Melamed S. Israely T. (2022). Elevation in sphingolipid upon SARS-CoV-2 infection: possible implications for COVID-19 pathology. Life Sci. alliance 5 (1), e202101168. doi: 10.26508/lsa.202101168 34764206 PMC8605327

[B25] Wang Z. Hu X. Li Z. Tu C. Wang Y. Pang P.. (2020). Effect of SARS-CoV-2 infection on the microbial composition of upper airway. Infect. Drug Resist. 13, 2637–2640. doi: 10.2147/IDR.S259984 32801801 PMC7406177

[B26] Wang X. Tan L. Wang X. Liu W. Lu Y. Cheng L.. (2020). Comparison of nasopharyngeal and oropharyngeal swabs for SARS-CoV-2 detection in 353 patients received tests with both specimens simultaneously. Int. J. Infect. Dis. 94, 107–109. doi: 10.1016/j.ijid.2020.04.023 32315809 PMC7166099

[B27] Wood D. E. Lu J. Langmead B. (2019). Improved metagenomic analysis with kraken 2. Genome Biol. 20 (1), 257. doi: 10.1186/s13059-019-1891-0 31779668 PMC6883579

[B28] Wu J. Liu J. Zhao X. Liu C. Wang W. Wang D.. (2020). Clinical characteristics of imported cases of coronavirus disease 2019 (COVID-19) in jiangsu province: a multicenter descriptive study. Clin. Infect. Dis. 71 (15), 706–712. doi: 10.1093/cid/ciaa199 32109279 PMC7108195

[B29] Xie C. Mao X. Huang J. Ding Y. Wu J. Dong S.. (2011). KOBAS 2.0: a web server for annotation and identification of enriched pathways and diseases. Nucleic Acids Res. 39 (Web Server issue), W316–W322. doi: 10.1093/nar/gkr483 21715386 PMC3125809

[B30] Ye S. Lu C. Qiu Y. Zheng H. Ge X. Wu A.. (2022). An atlas of human viruses provides new insights into diversity and tissue tropism of human viruses. Bioinformatics 38 (11), 3087–3093. doi: 10.1093/bioinformatics/btac275 35435220

[B31] Ye J. McGinnis S. Madden T. L. (2006). BLAST: improvements for better sequence analysis. Nucleic Acids Res. 34 (suppl_2), W6–W9. doi: 10.1093/nar/gkl164 16845079 PMC1538791

[B32] Yuan X. Li X. Kang Y. Pang L. Hei G. Zhang X.. (2022). Gut mycobiota dysbiosis in drug-naive, first-episode schizophrenia. Schizophr. Res. 250, 76–86. doi: 10.1016/j.schres.2022.10.011 36370535

[B33] Zhu W. Lomsadze A. Borodovsky M. (2010). Ab initio gene identification in metagenomic sequences. Nucleic Acids Res. 38 (12), e132–e132. doi: 10.1093/nar/gkq275 20403810 PMC2896542

