# Supplementary material for: Metatranscriptomic analysis revealed Prevotella as a potential biomarker of oropharyngeal microbiomes in SARS-CoV-2 infection
Source: Front Cell Infect Microbiol. 2023 Jun 2;13:1161763. doi: 10.3389/fcimb.2023.1161763 (PMC10272425; doi:10.3389/fcimb.2023.1161763)
Supplement: Supplementary file 1 [file DataSheet_1.docx]

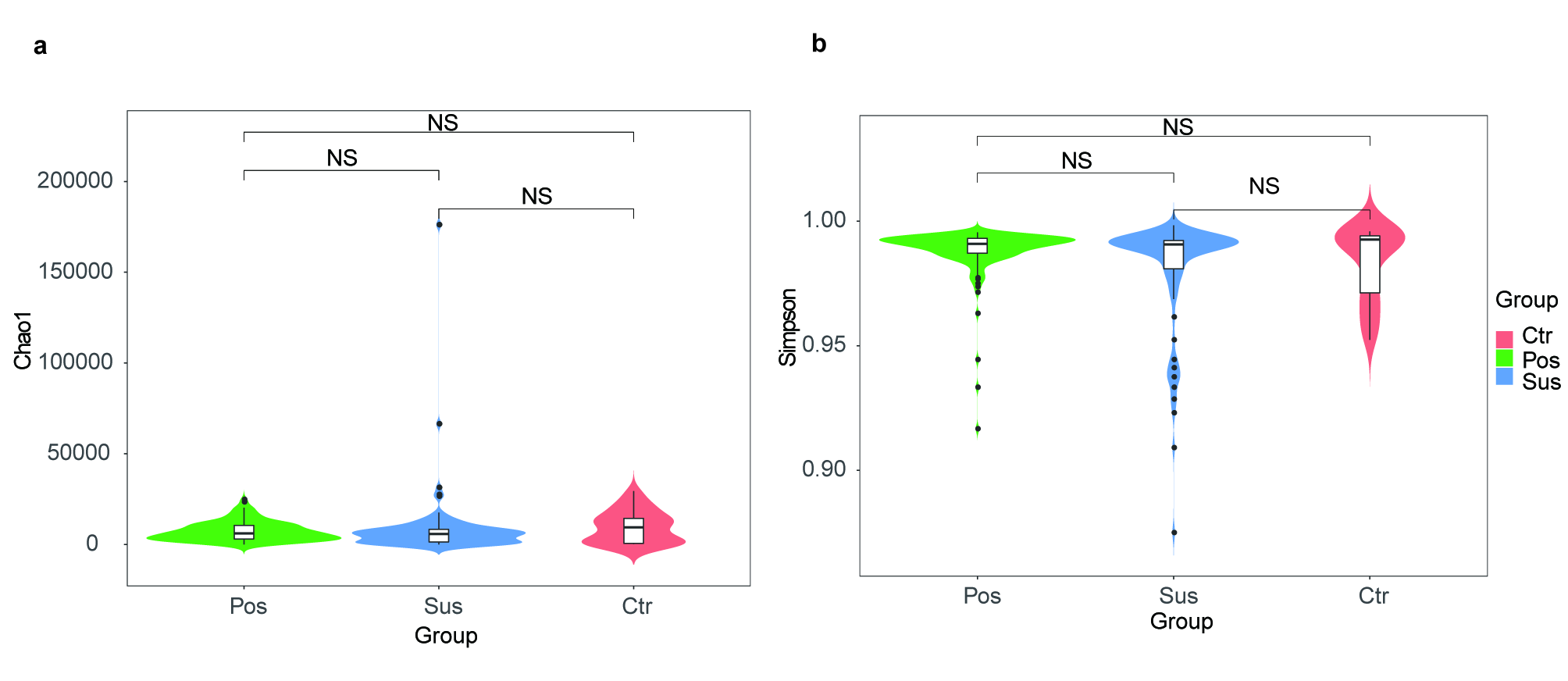


**Figure S1**. There were little changes in alpha diversity among Pos, Sus and Ctr, including **a** Chao1 index (*P* > 0.05), **b** Simpson index (*P* > 0.05).NS: not significant.


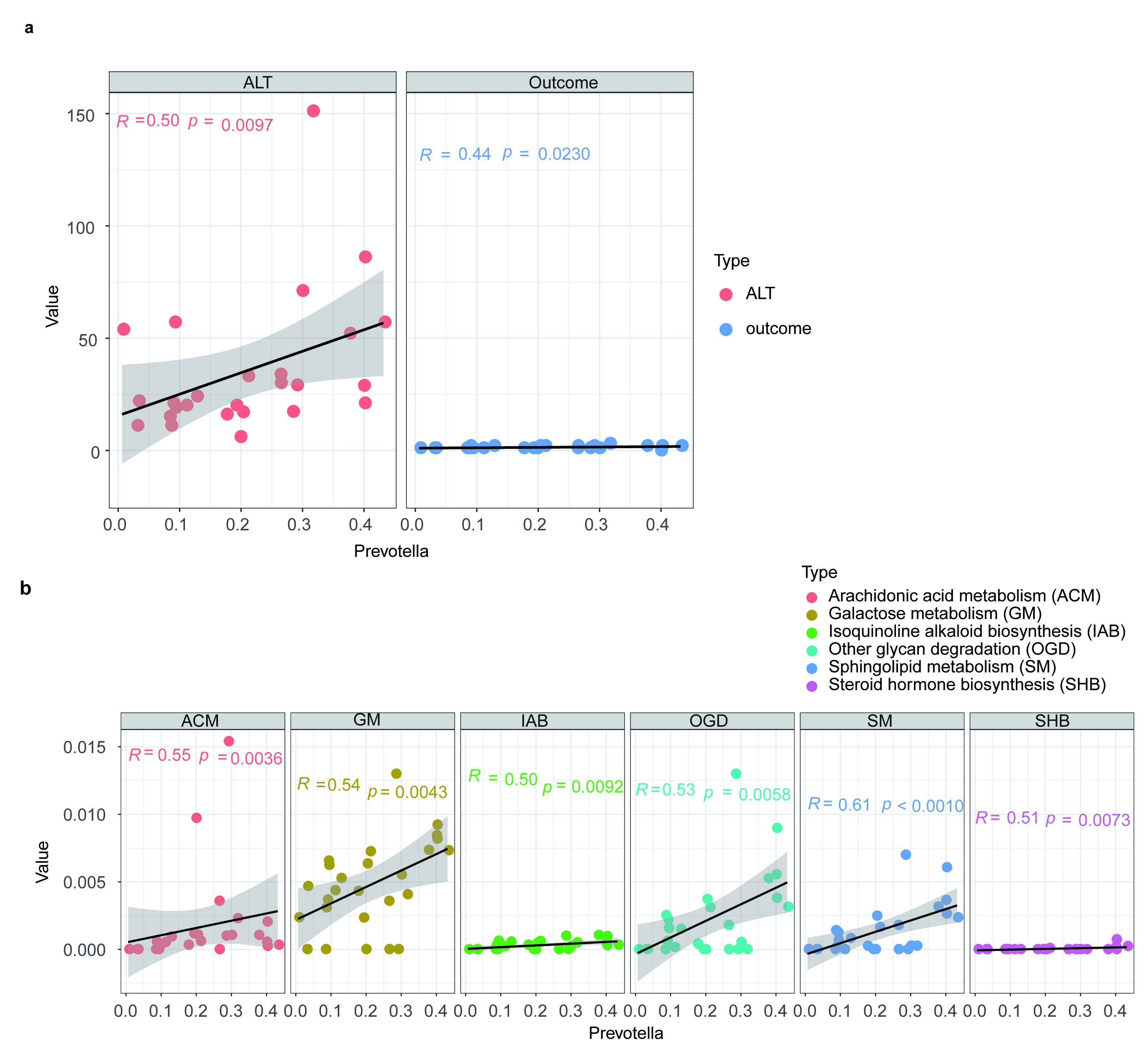


**Figure S2**. Potential function of *Prevotella* in *SARS-CoV-2* infection. **a** Spearman’s correlation between the relative abundance of *Prevotella* and the value of clinical indicators. **b** Spearman’s correlation between the relative abundance of *Prevotella* and the number of *Prevotella* gene involved in these pathways.

**
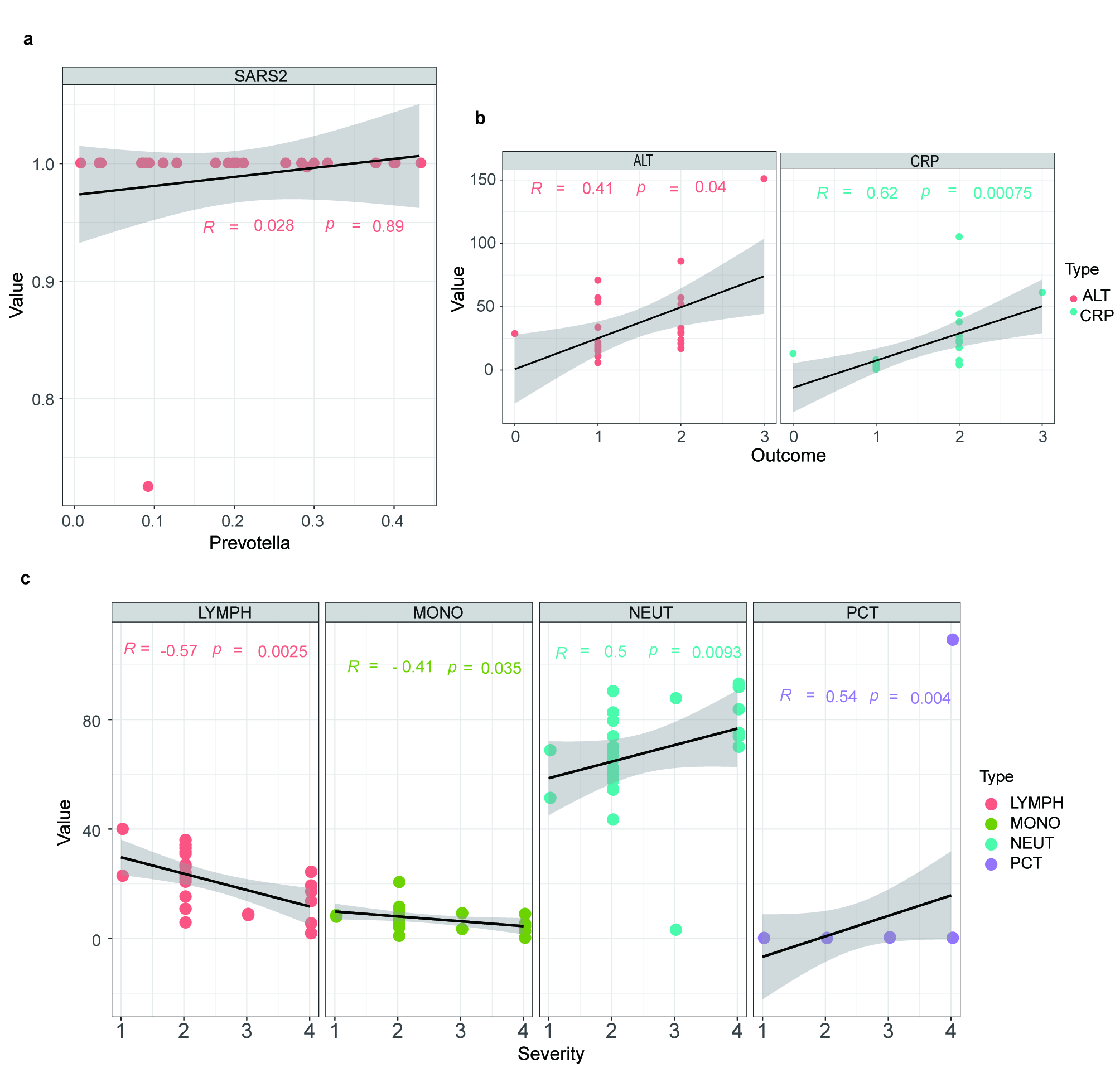
**

**Figure S3**. Some important correlations. **a** Correlations between the relative abundance of *Prevotella* and the relative abundance of *SARS-CoV-2*. **b** Correlations between the value of [Alanine transaminase](javascript:;) (ALT), [high-sensitivity C-reactive protein](javascript:;) (CRP) and the value of Outcome. **c** Correlations between the percentage of lymphocytes (LYMPH), monocytes (MONO), neutrophils (NEUT) , the value of Procalcitonin (PCT) and the value of Severity. Note: the higher the value of outcome, the worse the prognosis (1: recovery, 2: Improved or continue to stay, 3: Death); the higher the value of Severity, the more serious it is (1: mild, 2: moderate, 3: severe, 4: critical).

**Table S1.** The P-values of bacteria among Pos, Sus and Ctr group.

| **Bacteria** | **Pos VS Sus** | **Pos VS Ctr** | **Sus VS Ctr** |
| --- | --- | --- | --- |
| *Fusobacterium* | 6.73E-01 | 1.15E-04 | 0.005517113 |
| *Aggregatibacter* | 1.11E-02 | 1.10E-04 | 0.056195528 |
| *Aequorivita* | 3.48E-02 | 5.33E-02 | 0.001727399 |
| *Candidatus_Pelagibacter* | 2.99E-07 | 9.80E-04 | 0.336815295 |
| *Prevotella* | 7.00E-06 | 4.21E-07 | 0.047929355 |
| *Leptotrichia* | 7.00E-17 | 1.07E-08 | 0.913058507 |
| *Porphyromonas* | 1.13E-03 | 8.80E-07 | 0.011902068 |
| *Alloprevotella* | 2.25E-10 | 2.31E-05 | 0.832579813 |
| *Treponema* | 1.50E-09 | 1.28E-08 | 0.08917203 |
| *Capnocytophaga* | 1.78E-04 | 1.43E-05 | 0.106420559 |
| *Lautropia* | 4.18E-05 | 3.77E-06 | 0.082683074 |
| *Selenomonas* | 3.57E-07 | 2.82E-04 | 0.82906122 |
| *Glaesserella* | 2.21E-04 | 2.99E-01 | 0.068719102 |
| *Tannerella* | 3.81E-05 | 1.18E-05 | 0.212238323 |
| *Parabacteroides* | 1.48E-04 | 2.48E-03 | 0.643662107 |
| *Actinomyces* | 7.68E-04 | 4.85E-02 | 0.90812631 |
| *Dolosigranulum* | 1.47E-04 | 8.25E-09 | 0.004107993 |
| *Mycoplasma* | 2.21E-01 | 1.40E-02 | 0.0003409 |
| *Schaalia* | 1.75E-02 | 1.50E-02 | 0.508197713 |
| *Kytococcus* | 2.45E-01 | 2.41E-03 | 0.043889695 |

**Table S2.** The P-values of fungi among Pos, Sus and Ctr group.

| **Fungi** | **Pos VS Sus** | **Pos VS Ctr** | **Sus VS Ctr** |
| --- | --- | --- | --- |
| *Malassezia* | 0.001583376 | 1.95E-02 | 7.75E-01 |
| *Phaeoacremonium* | 0.763793979 | 1.88E-03 | 4.60E-03 |
| *Botrytis* | 0.953682948 | 1.39E-02 | 1.82E-02 |
| *Endocarpon* | 0.776107746 | 2.10E-03 | 4.38E-03 |
| *Aspergillus* | 0.031971929 | 2.89E-04 | 2.01E-02 |
| *Saccharomyces* | 0.092866359 | 1.76E-02 | 7.33E-05 |
| *Melampsora* | 0.003780361 | 6.37E-01 | 4.42E-02 |
| *Yarrowia* | 0.008073644 | 6.61E-02 | 8.27E-01 |
| *Debaryomyces* | 0.507710508 | 4.38E-03 | 2.39E-03 |
| *Lobosporangium* | 0.3607643 | 2.14E-03 | 1.05E-02 |
| *Marssonina* | 0.651913631 | 6.44E-03 | 1.71E-02 |
| *Pestalotiopsis* | 0.201259653 | 9.51E-03 | 7.67E-02 |
| *Daldinia* | 0.955882429 | 3.12E-03 | 4.01E-03 |
| *Clavispora* | 0.062061608 | 6.26E-04 | 5.05E-05 |
| *Pseudogymnoascus* | 0.105089005 | 4.63E-03 | 4.37E-05 |
| *Komagataella* | 0.07933889 | 2.00E-03 | 1.28E-05 |
| *Lachancea* | 0.702822766 | 1.60E-04 | 2.33E-03 |
| *Pseudomicrostroma* | 0.037325202 | 1.84E-05 | 2.77E-02 |
| *Schizosaccharomyces* | 0.372847382 | 8.82E-03 | 1.28E-03 |
| *Tremella* | 0.317817429 | 2.25E-05 | 2.24E-03 |
| *Alternaria* | 0.056567067 | 1.78E-01 | 4.69E-03 |
| *Chaetomium* | 0.709902224 | 1.93E-03 | 1.06E-02 |
| *Sugiyamaella* | 0.945063749 | 1.18E-04 | 7.46E-04 |

**Table S3.** The P-values of viruses among Pos, Sus and Ctr group.

| **Viruses** | **Pos VS Sus** | **Pos VS Ctr** | **Sus VS Ctr** |
| --- | --- | --- | --- |
| *Human alphaherpesvirus 1* | 9.66E-02 | 0.460486 | 0.1533823 |
| *Human alphaherpesvirus 2* | 4.09E-01 | 0.607343 | NA |
| *Human betaherpesvirus 5* | 2.33E-01 | NA | 0.5374695 |
| *Human betaherpesvirus 7* | 4.09E-01 | 0.607343 | NA |
| *Human gammaherpesvirus 4* | 3.06E-01 | 0.434572 | 0.18413 |
| *Human mastadenovirus C* | 4.09E-01 | 0.607343 | NA |
| *Human metapneumovirus* | 9.01E-02 | NA | 0.3753798 |
| *Porcine type-C oncovirus* | 1.49E-01 | 0.36269 | NA |
| *Rhinovirus A* | NA | 0.059539 | 0.1175062 |
| *Rhinovirus C* | NA | 0.059539 | 0.1175062 |
| SARS-CoV-2 | 7.38E-06 | 0.003889 | NA |

**Table S4. The read counts of the 290 involved sample.**

| Groups | Samples | Total_clean_reads | Human_map_reads | Unmap_reads |
| --- | --- | --- | --- | --- |
| Pos | 1-U1 | 18,523,131 | 462,098 | 18,061,033 |
| Pos | 106 | 18,933,214 | 11,777,016 | 7,156,198 |
| Pos | 111-U24 | 18,470,030 | 13,498,039 | 4,971,991 |
| Pos | 115-U28 | 19,166,478 | 6,683,445 | 12,483,033 |
| Pos | 118-1-U28 | 22,442,846 | 12,212,228 | 10,230,618 |
| Pos | 119 | 14,878,405 | 12,719,433 | 2,158,972 |
| Pos | 121-U34 | 15,525,879 | 2,493,226 | 13,032,653 |
| Pos | 124-U37 | 13,588,704 | 11,151,726 | 2,436,978 |
| Pos | 132-1-U7 | 21,830,156 | 3,997,341 | 17,832,815 |
| Pos | 133-U46 | 17,347,841 | 1,787,154 | 15,560,687 |
| Pos | 134-U47 | 16,092,928 | 1,511,923 | 14,581,005 |
| Pos | 137-1-U9 | 40,274,940 | 7,339,270 | 32,935,670 |
| Pos | 14-U4 | 20,795,955 | 12,766,798 | 8,029,157 |
| Pos | 141-1-U12 | 37,693,821 | 16,247,621 | 21,446,200 |
| Pos | 143-1-U14 | 20,504,777 | 7,819,831 | 12,684,946 |
| Pos | 144-1-U15 | 16,458,129 | 4,447,015 | 12,011,114 |
| Pos | 145-1-U16 | 17,953,080 | 219,867 | 17,733,213 |
| Pos | 148-1-U19 | 12,578,301 | 2,643,753 | 9,934,548 |
| Pos | 152-1-U22 | 23,406,166 | 19,392,043 | 4,014,123 |
| Pos | 159-1-U19 | 19,174,028 | 1,549,016 | 17,625,012 |
| Pos | 16-U6 | 20,487,328 | 1,414,652 | 19,072,676 |
| Pos | 160-1-U20 | 15,969,283 | 2,750,287 | 13,218,996 |
| Pos | 161-1-U21 | 18,476,989 | 8,655,065 | 9,821,924 |
| Pos | 162-1-U22 | 16,961,233 | 315,159 | 16,646,074 |
| Pos | 163-1-U23 | 22,213,816 | 389,508 | 21,824,308 |
| Pos | 165-1-U7 | 12,864,317 | 6,694,720 | 6,169,597 |
| Pos | 170 | 17,129,261 | 3,405,654 | 13,723,607 |
| Pos | 183 | 20,683,759 | 554,737 | 20,129,022 |
| Pos | 19-U8 | 18,263,705 | 9,632,479 | 8,631,226 |
| Pos | 192-1-U31 | 24,392,172 | 15,232,206 | 9,159,966 |
| Pos | 197-1-U36 | 15,502,050 | 1,432,504 | 14,069,546 |
| Pos | 200-1-U14 | 21,628,608 | 483,489 | 21,145,119 |
| Pos | 201-1-U15 | 18,940,049 | 5,640,718 | 13,299,331 |
| Pos | 202-1-U16 | 23,263,020 | 1,710,294 | 21,552,726 |
| Pos | 209-1-U23 | 15,184,163 | 8,162,043 | 7,022,120 |
| Pos | 20R001970-1-U2 | 19,603,389 | 16,818,083 | 2,785,306 |
| Pos | 20R002106-1-U8 | 14,898,155 | 13,581,996 | 1,316,159 |
| Pos | 213-1-U26 | 13,763,039 | 3,995,447 | 9,767,592 |
| Pos | 217-1-U28 | 21,845,946 | 20,622,741 | 1,223,205 |
| Pos | 37-U14 | 20,582,752 | 2,996,437 | 17,586,315 |
| Pos | 41-U17 | 29,224,646 | 12,912,713 | 16,311,933 |
| Pos | 4233 | 18,820,064 | 7,956,312 | 10,863,752 |
| Pos | 5032-Re | 23,573,259 | 22,236,065 | 1,337,194 |
| Pos | 55-U9 | 14,463,487 | 893,888 | 13,569,599 |
| Pos | 72 | 15,598,599 | 14,310,839 | 1,287,760 |
| Pos | 75-U15 | 16,510,557 | 569,093 | 15,941,464 |
| Pos | 83-1-U26 | 24,623,614 | 6,991,045 | 17,632,569 |
| Pos | 9002 | 25,990,177 | 12,525,518 | 13,464,659 |
| Pos | 9027 | 13,604,539 | 11,443,621 | 2,160,918 |
| Pos | 9052 | 18,599,509 | 17,367,801 | 1,231,708 |
| Pos | 9056 | 19,710,295 | 17,108,063 | 2,602,232 |
| Pos | 9075 | 26,063,574 | 24,665,924 | 1,397,650 |
| Pos | 9133 | 23,531,354 | 19,755,099 | 3,776,255 |
| Pos | 9186 | 22,505,723 | 13,946,267 | 8,559,456 |
| Pos | 9299-1-U8 | 19,574,130 | 8,076,837 | 11,497,293 |
| Pos | 9302-1-U9 | 19,837,762 | 4,232,324 | 15,605,438 |
| Pos | 9303-1-U10 | 16,341,213 | 15,263,252 | 1,077,961 |
| Pos | 9309-1-U11 | 18,831,169 | 4,493,064 | 14,338,105 |
| Pos | 9511-2 | 20,485,478 | 14,795,659 | 5,689,819 |
| Pos | 9512-2 | 30,832,648 | 15,769,794 | 15,062,854 |
| Pos | 9514-3 | 22,229,591 | 20,230,119 | 1,999,472 |
| Pos | 9520-1-U35 | 24,049,599 | 552,561 | 23,497,038 |
| Pos | 9521-1-U15 | 18,854,619 | 1,559,827 | 17,294,792 |
| Pos | 9522-1-U36 | 22,073,520 | 697,500 | 21,376,020 |
| Pos | 9525-1-U12 | 20,390,996 | 6,260,086 | 14,130,910 |
| Pos | 9527-1-U16 | 17,077,102 | 1,482,144 | 15,594,958 |
| Pos | 9529-1-U17 | 18,562,247 | 626,997 | 17,935,250 |
| Pos | CV1 | 17,639,425 | 15,524,108 | 2,115,317 |
| Pos | CV10 | 17,175,442 | 3,931,543 | 13,243,899 |
| Pos | CV11 | 18,260,871 | 10,845,488 | 7,415,383 |
| Pos | CV12 | 17,390,345 | 10,189,137 | 7,201,208 |
| Pos | CV125-1-U13 | 23,467,294 | 11,893,284 | 11,574,010 |
| Pos | CV127-1-U14 | 20,144,153 | 18,294,540 | 1,849,613 |
| Pos | CV128-1-U14 | 20,374,891 | 13,961,372 | 6,413,519 |
| Pos | CV13 | 17,743,105 | 6,946,497 | 10,796,608 |
| Pos | CV14 | 18,305,136 | 10,770,547 | 7,534,589 |
| Pos | CV15 | 17,766,250 | 17,092,809 | 673,441 |
| Pos | CV16 | 16,884,651 | 11,614,877 | 5,269,774 |
| Pos | CV17 | 22,391,574 | 17,644,339 | 4,747,235 |
| Pos | CV2-1-U19 | 16,274,390 | 9,540,829 | 6,733,561 |
| Pos | CV20 | 16,429,065 | 13,898,476 | 2,530,589 |
| Pos | CV24 | 20,645,733 | 14,756,791 | 5,888,942 |
| Pos | CV241-1-U21 | 26,739,909 | 25,924,307 | 815,602 |
| Pos | CV242-1-U15 | 23,067,468 | 22,445,218 | 622,250 |
| Pos | CV243-1-U22 | 14,924,575 | 14,062,297 | 862,278 |
| Pos | CV244-1-U23 | 22,088,441 | 9,254,605 | 12,833,836 |
| Pos | CV245-1-U24 | 21,160,328 | 20,512,043 | 648,285 |
| Pos | CV246-1-U25 | 22,759,916 | 22,157,617 | 602,299 |
| Pos | CV247-1-U26 | 22,717,885 | 17,132,936 | 5,584,949 |
| Pos | CV25 | 16,427,062 | 12,183,877 | 4,243,185 |
| Pos | CV252-1-U28 | 26,447,752 | 3,147,475 | 23,300,277 |
| Pos | CV26 | 18,343,082 | 9,844,367 | 8,498,715 |
| Pos | CV269-1-1-U29 | 17,632,856 | 14,440,696 | 3,192,160 |
| Pos | CV270-1-U31 | 18,465,538 | 5,308,101 | 13,157,437 |
| Pos | CV28-1-U12 | 25,396,833 | 19,773,328 | 5,623,505 |
| Pos | CV29-1-U8 | 18,364,540 | 14,393,070 | 3,971,470 |
| Pos | CV3 | 17,823,731 | 11,551,373 | 6,272,358 |
| Pos | CV30-1-U13 | 30,371,618 | 12,986,256 | 17,385,362 |
| Pos | CV31-1-U14 | 22,463,019 | 18,904,535 | 3,558,484 |
| Pos | CV33-1-U9 | 20,487,247 | 13,620,767 | 6,866,480 |
| Pos | CV34 | 43,239,914 | 23,267,071 | 19,972,843 |
| Pos | CV35 | 25,814,754 | 18,458,003 | 7,356,751 |
| Pos | CV4 | 17,199,246 | 3,118,723 | 14,080,523 |
| Pos | CV41 | 38,150,343 | 8,724,193 | 29,426,150 |
| Pos | CV42 | 39,977,253 | 18,128,333 | 21,848,920 |
| Pos | CV43 | 27,411,782 | 18,600,132 | 8,811,650 |
| Pos | CV44-1-U15 | 25,005,348 | 1,819,497 | 23,185,851 |
| Pos | CV45-1-U20 | 17,545,818 | 12,583,961 | 4,961,857 |
| Pos | CV46-1-U11 | 23,168,155 | 9,958,805 | 13,209,350 |
| Pos | CV50-1-U16 | 23,208,048 | 9,397,697 | 13,810,351 |
| Pos | CV56-1-U12 | 20,515,623 | 7,886,553 | 12,629,070 |
| Pos | CV7 | 18,289,807 | 4,196,467 | 14,093,340 |
| Pos | CV8 | 18,816,035 | 2,819,379 | 15,996,656 |
| Pos | CV9 | 17,433,507 | 6,602,780 | 10,830,727 |
| Pos | HX-20200208-21-U21 | 17,357,136 | 12,990,649 | 4,366,487 |
| Pos | HX-20200208-22-U22 | 14,700,434 | 2,702,448 | 11,997,986 |
| Pos | HX-20200208-23-U23 | 15,432,730 | 8,271,801 | 7,160,929 |
| Pos | HX-20200216-10-1-U10 | 17,896,721 | 14,931,890 | 2,964,831 |
| Pos | HX-20200216-11-1-U11 | 19,819,669 | 14,792,613 | 5,027,056 |
| Pos | HX-20200216-12-1-U12 | 17,427,264 | 12,810,666 | 4,616,598 |
| Pos | HX-20200216-13-1-U13 | 19,134,947 | 14,928,081 | 4,206,866 |
| Pos | HX-20200216-15-1-U15 | 19,883,209 | 1,803,954 | 18,079,255 |
| Pos | HX-20200216-16-1-U16 | 17,788,964 | 15,929,747 | 1,859,217 |
| Pos | HX-20200216-17-1-U17 | 13,934,611 | 9,589,474 | 4,345,137 |
| Pos | HX-20200216-18-1-U18 | 25,365,785 | 20,596,617 | 4,769,168 |
| Pos | HX-20200216-19-1-U19 | 22,651,751 | 6,762,422 | 15,889,329 |
| Pos | HX-20200216-3-1-U3 | 16,621,807 | 15,761,691 | 860,116 |
| Pos | HX-20200216-4-1-U4 | 17,783,139 | 13,369,187 | 4,413,952 |
| Pos | HX-20200216-5-1-U5 | 18,668,087 | 17,709,005 | 959,082 |
| Pos | HX-20200216-6-1-U6 | 19,138,839 | 18,113,039 | 1,025,800 |
| Pos | HX-20200216-7-1-U7 | 21,474,856 | 6,862,512 | 14,612,344 |
| Pos | HX-20200216-8-1-U8 | 19,919,665 | 17,582,182 | 2,337,483 |
| Pos | HX-20200216-9-1-U9 | 20,131,306 | 17,897,599 | 2,233,707 |
| Pos | P1-1-U1 | 22,328,204 | 19,764,750 | 2,563,454 |
| Pos | P13-1-U35 | 17,405,966 | 1,028,588 | 16,377,378 |
| Pos | P14-1-U12 | 19,547,019 | 546,526 | 19,000,493 |
| Pos | P2-1-U2 | 24,154,622 | 8,347,388 | 15,807,234 |
| Pos | P3-1-U3 | 19,441,182 | 1,778,002 | 17,663,180 |
| Pos | P4-1-U4 | 22,697,719 | 14,304,414 | 8,393,305 |
| Pos | P5-1-U5 | 18,941,024 | 1,314,201 | 17,626,823 |
| Pos | P7-1-U7 | 22,117,390 | 1,980,584 | 20,136,806 |
| Pos | P8-1-U8 | 21,259,229 | 4,067,677 | 17,191,552 |
| Pos | R10-1-U14 | 12,913,770 | 3,498,684 | 9,415,086 |
| Pos | WYC-1-U22 | 21,629,939 | 5,802,530 | 15,827,409 |
| Sus | 9349 | 12,691,845 | 1,170,793 | 11,521,052 |
| Sus | 9614 | 12,578,297 | 1,807,304 | 10,770,993 |
| Sus | H-7-107 | 13,572,294 | 8,162,822 | 5,409,472 |
| Sus | H-7-108-1-U7 | 14,443,950 | 4,424,111 | 10,019,839 |
| Sus | H-7-109-1-U8 | 14,506,525 | 120,311 | 14,386,214 |
| Sus | H-7-110-1-U9 | 25,769,747 | 819,324 | 24,950,423 |
| Sus | H-7-111-1-U10 | 21,122,362 | 10,158,007 | 10,964,355 |
| Sus | H-7-112-1-U11 | 18,131,040 | 2,602,273 | 15,528,767 |
| Sus | HX-20200216-20-1-U1 | 19,029,132 | 17,504,414 | 1,524,718 |
| Sus | HX-20200216-21-1-U2 | 19,307,784 | 11,677,734 | 7,630,050 |
| Sus | HX-20200216-22-1-U3 | 16,937,826 | 16,103,515 | 834,311 |
| Sus | HX-20200216-23-1-U4 | 17,919,456 | 5,751,363 | 12,168,093 |
| Sus | HX-20200216-24-1-U5 | 19,212,480 | 11,012,208 | 8,200,272 |
| Sus | HX-20200216-25-1-U6 | 24,959,523 | 20,621,291 | 4,338,232 |
| Sus | HX-20200216-26-1-U7 | 21,479,416 | 19,441,453 | 2,037,963 |
| Sus | HX-20200216-27-1-U8 | 19,036,656 | 11,951,749 | 7,084,907 |
| Sus | HX-20200216-29-1-U10 | 16,280,314 | 2,524,712 | 13,755,602 |
| Sus | HX-20200216-31-1-U12 | 18,463,893 | 6,818,930 | 11,644,963 |
| Sus | HX-20200216-32-1-U13 | 21,702,106 | 6,634,493 | 15,067,613 |
| Sus | HX-20200216-34-1-U15 | 19,242,105 | 18,770,771 | 471,334 |
| Sus | HX-20200216-36-1-U17 | 31,754,111 | 821,824 | 30,932,287 |
| Sus | HX-20200216-37-1-U18 | 21,116,893 | 20,651,651 | 465,242 |
| Sus | HX-20200216-41-1-U22 | 19,262,216 | 16,711,682 | 2,550,534 |
| Sus | HX-7-1-U1 | 15,759,809 | 4,020,295 | 11,739,514 |
| Sus | HX-7-100-1-U39 | 14,292,929 | 3,998,765 | 10,294,164 |
| Sus | HX-7-101-1-U40 | 17,118,529 | 4,466,961 | 12,651,568 |
| Sus | HX-7-102-1-U41 | 17,112,738 | 3,124,544 | 13,988,194 |
| Sus | HX-7-103-1-U42 | 15,391,584 | 1,446,213 | 13,945,371 |
| Sus | HX-7-104-1-U43 | 16,636,563 | 4,670,460 | 11,966,103 |
| Sus | HX-7-105-1-U44 | 16,811,439 | 11,516,147 | 5,295,292 |
| Sus | HX-7-106 | 13,325,055 | 7,023,298 | 6,301,757 |
| Sus | HX-7-11-U11 | 18,877,661 | 17,236,662 | 1,640,999 |
| Sus | HX-7-12-U12 | 18,142,899 | 16,734,397 | 1,408,502 |
| Sus | HX-7-13-U13 | 17,686,269 | 1,898,779 | 15,787,490 |
| Sus | HX-7-14-U14 | 19,158,690 | 3,509,975 | 15,648,715 |
| Sus | HX-7-17-U17 | 20,268,793 | 19,317,110 | 951,683 |
| Sus | HX-7-18-U18 | 19,734,077 | 19,160,725 | 573,352 |
| Sus | HX-7-2-U2 | 16,792,515 | 6,665,215 | 10,127,300 |
| Sus | HX-7-21-U21 | 19,434,987 | 18,669,730 | 765,257 |
| Sus | HX-7-22-U22 | 15,477,891 | 2,959,508 | 12,518,383 |
| Sus | HX-7-23 | 11,792,539 | 5,458,268 | 6,334,271 |
| Sus | HX-7-25-U3 | 14,932,094 | 847,004 | 14,085,090 |
| Sus | HX-7-26-U4 | 14,251,011 | 4,541,118 | 9,709,893 |
| Sus | HX-7-27-U5 | 13,576,923 | 969,616 | 12,607,307 |
| Sus | HX-7-28-U6 | 16,363,567 | 717,688 | 15,645,879 |
| Sus | HX-7-29-U7 | 17,676,841 | 5,538,817 | 12,138,024 |
| Sus | HX-7-30-U8 | 18,224,831 | 1,490,474 | 16,734,357 |
| Sus | HX-7-31-U9 | 16,623,861 | 1,182,737 | 15,441,124 |
| Sus | HX-7-34-U12 | 18,400,817 | 3,399,197 | 15,001,620 |
| Sus | HX-7-35-U13 | 15,790,116 | 1,629,814 | 14,160,302 |
| Sus | HX-7-38-U16 | 17,021,107 | 3,716,331 | 13,304,776 |
| Sus | HX-7-39-U17 | 19,046,982 | 7,232,064 | 11,814,918 |
| Sus | HX-7-4-U4 | 19,899,227 | 7,092,831 | 12,806,396 |
| Sus | HX-7-40-U18 | 20,237,451 | 4,063,755 | 16,173,696 |
| Sus | HX-7-41-U19 | 16,852,807 | 5,630,907 | 11,221,900 |
| Sus | HX-7-42-U20 | 20,109,836 | 19,349,683 | 760,153 |
| Sus | HX-7-43-U21 | 21,059,937 | 36,394 | 21,023,543 |
| Sus | HX-7-44-U22 | 15,804,079 | 3,825,817 | 11,978,262 |
| Sus | HX-7-47-U25 | 15,940,721 | 15,295,108 | 645,613 |
| Sus | HX-7-48-U26 | 14,074,382 | 11,769,001 | 2,305,381 |
| Sus | HX-7-49-U27 | 10,036,604 | 9,321,794 | 714,810 |
| Sus | HX-7-51-U25 | 39,602,353 | 34,412,931 | 5,189,422 |
| Sus | HX-7-53-U27 | 27,447,158 | 9,927,161 | 17,519,997 |
| Sus | HX-7-54-U28 | 41,215,912 | 33,251,062 | 7,964,850 |
| Sus | HX-7-55-U29 | 45,125,924 | 38,481,028 | 6,644,896 |
| Sus | HX-7-56-U30 | 43,461,952 | 1,282,244 | 42,179,708 |
| Sus | HX-7-57-U31 | 15,632,524 | 2,041,746 | 13,590,778 |
| Sus | HX-7-58-U32 | 12,952,702 | 830,108 | 12,122,594 |
| Sus | HX-7-59-U33 | 13,541,031 | 3,019,324 | 10,521,707 |
| Sus | HX-7-6-U6 | 18,468,116 | 1,169,696 | 17,298,420 |
| Sus | HX-7-60-U34 | 14,064,844 | 4,454,315 | 9,610,529 |
| Sus | HX-7-61-U5 | 13,690,021 | 10,760,530 | 2,929,491 |
| Sus | HX-7-62-U6 | 21,520,323 | 20,339,086 | 1,181,237 |
| Sus | HX-7-63-U7 | 20,628,768 | 12,245,349 | 8,383,419 |
| Sus | HX-7-64-U8 | 25,289,531 | 22,211,793 | 3,077,738 |
| Sus | HX-7-65-U9 | 18,148,012 | 14,898,239 | 3,249,773 |
| Sus | HX-7-66-U10 | 22,220,209 | 12,445,071 | 9,775,138 |
| Sus | HX-7-67-U11 | 21,610,244 | 17,782,591 | 3,827,653 |
| Sus | HX-7-68 | 13,461,957 | 3,418,101 | 10,043,856 |
| Sus | HX-7-69-U13 | 18,298,158 | 13,578,633 | 4,719,525 |
| Sus | HX-7-7-U7 | 21,590,270 | 831,923 | 20,758,347 |
| Sus | HX-7-70-U14 | 19,924,858 | 18,891,650 | 1,033,208 |
| Sus | HX-7-71 | 11,584,908 | 9,586,166 | 1,998,742 |
| Sus | HX-7-72-U16 | 13,856,915 | 12,768,864 | 1,088,051 |
| Sus | HX-7-73-U17 | 26,178,906 | 18,888,247 | 7,290,659 |
| Sus | HX-7-74-U18 | 22,456,852 | 20,562,549 | 1,894,303 |
| Sus | HX-7-75 | 10,989,608 | 10,100,580 | 889,028 |
| Sus | HX-7-76-1-U2 | 18,871,171 | 17,664,247 | 1,206,924 |
| Sus | HX-7-77-1-U3 | 19,476,202 | 9,436,222 | 10,039,980 |
| Sus | HX-7-8-U8 | 20,386,942 | 19,096,705 | 1,290,237 |
| Sus | HX-7-82-1-U8 | 234,018,276 | 45,559,126 | 188,459,150 |
| Sus | HX-7-83-1-U9 | 16,887,074 | 16,050,043 | 837,031 |
| Sus | HX-7-84-1-U10 | 16,401,795 | 9,829,144 | 6,572,651 |
| Sus | HX-7-85-1-U11 | 18,520,867 | 5,081,681 | 13,439,186 |
| Sus | HX-7-9-U9 | 19,820,780 | 18,480,754 | 1,340,026 |
| Sus | HX-7-92-1-U18 | 18,350,525 | 2,645,069 | 15,705,456 |
| Sus | HX-7-94-1-U20 | 17,045,935 | 2,937,005 | 14,108,930 |
| Sus | HX-7-95-1-U21 | 16,608,191 | 389,932 | 16,218,259 |
| Sus | HX-7-96-1-U22 | 15,185,924 | 14,750,843 | 435,081 |
| Sus | HX-7-99-1-U38 | 16,534,446 | 3,681,795 | 12,852,651 |
| Ctr | 2248-U42 | 18,744,799 | 944,950 | 17,799,849 |
| Ctr | 2250-U30 | 24,415,426 | 855,629 | 23,559,797 |
| Ctr | 2253-1-U43 | 27,874,654 | 166,004 | 27,708,650 |
| Ctr | 2265-1-U24 | 16,142,032 | 967,542 | 15,174,490 |
| Ctr | 2270-U31 | 29,618,320 | 386,206 | 29,232,114 |
| Ctr | 2275-U32 | 30,505,105 | 994,336 | 29,510,769 |
| Ctr | 4242-U24 | 27,614,671 | 921,278 | 26,693,393 |
| Ctr | 4243-1-U36 | 15,792,156 | 2,588,294 | 13,203,862 |
| Ctr | 4246-1-U42 | 19,511,833 | 3,340,595 | 16,171,238 |
| Ctr | 4248-U25 | 28,941,490 | 1,318,476 | 27,623,014 |
| Ctr | 4249-1-U37 | 15,571,075 | 11,005,045 | 4,566,030 |
| Ctr | 4251-1-U38 | 37,757,928 | 10,863,240 | 26,894,688 |
| Ctr | 4252-1-U39 | 17,835,806 | 1,454,697 | 16,381,109 |
| Ctr | 4253-1-U40 | 33,195,820 | 1,386,738 | 31,809,082 |
| Ctr | 4254-1-U41 | 31,171,477 | 1,984,462 | 29,187,015 |
| Ctr | 4295-U33 | 15,053,300 | 4,852,118 | 10,201,182 |
| Ctr | 4312-U26 | 26,425,763 | 23,844,643 | 2,581,120 |
| Ctr | 4315-U27 | 37,492,260 | 13,262,380 | 24,229,880 |
| Ctr | 4324-U34 | 27,171,105 | 10,409,816 | 16,761,289 |
| Ctr | 4333-1-U39 | 31,773,463 | 17,712,967 | 14,060,496 |
| Ctr | 4372-1-U40 | 26,347,749 | 9,959,578 | 16,388,171 |
| Ctr | 4380-1-U15 | 30,853,958 | 2,294,084 | 28,559,874 |
| Ctr | 4393-U29 | 21,761,146 | 3,886,526 | 17,874,620 |
| Ctr | 4394-U28 | 33,642,727 | 4,354,221 | 29,288,506 |
| Ctr | 4408-U41 | 22,568,425 | 10,514,417 | 12,054,008 |
| Ctr | 7881-U36 | 32,751,157 | 418,646 | 32,332,511 |
| Ctr | 7882-1-U17 | 29,511,669 | 28,104,541 | 1,407,128 |
| Ctr | 7883-U37 | 30,363,568 | 28,701,910 | 1,661,658 |
| Ctr | 7884-U38 | 30,732,808 | 29,075,021 | 1,657,787 |
| Ctr | 7885-U39 | 25,127,801 | 24,019,024 | 1,108,777 |
| Ctr | 7886-U40 | 31,236,215 | 15,605,751 | 15,630,464 |
| Ctr | 7887-1-U18 | 30,512,358 | 29,439,121 | 1,073,237 |
| Ctr | 7888-1-U38 | 20,053,307 | 19,332,580 | 720,727 |
| Ctr | 7889-1-U35 | 27,793,819 | 26,949,199 | 844,620 |
| Ctr | 7892-1-U19 | 24,945,061 | 19,397,194 | 5,547,867 |
| Ctr | 7893-1-U20 | 30,868,027 | 29,772,354 | 1,095,673 |
| Ctr | 7894-1-U21 | 28,170,309 | 26,885,484 | 1,284,825 |
| Ctr | 7895-1-U22 | 29,165,798 | 28,157,615 | 1,008,183 |
| Ctr | 7896-1-U23 | 31,675,674 | 30,562,835 | 1,112,839 |
| Ctr | 7901-U35 | 30,505,239 | 29,352,412 | 1,152,827 |
| NC | NC1 | 645,649 | 99,968 | 545,681 |
| NC | NC2 | 6,409,819 | 912,474 | 5,497,345 |
| NC | NC3 | 14,421,389 | 5,089,277 | 9,332,112 |
| NC | NC4 | 11,764,432 | 1,543,727 | 10,220,705 |
| NC | NC5 | 650,867 | 114,628 | 536,239 |
| NC | NC6 | 27,200,744 | 1,179,993 | 26,020,751 |
